# Developmental changes of bodily self-consciousness in adolescent girls

**DOI:** 10.1038/s41598-024-61253-6

**Published:** 2024-05-17

**Authors:** Lisa Raoul, Cédric Goulon, Fabrice Sarlegna, Marie-Hélène Grosbras

**Affiliations:** 1 Aix Marseille Univ, CNRS, CRPN, Marseille, France; 2 Aix Marseille Univ, CNRS, ISM, Marseille, France

**Keywords:** Human behaviour, Sensorimotor processing, Sensory processing

## Abstract

The body and the self change markedly during adolescence, but how does bodily self-consciousness, the pre-reflexive experience of being a bodily subject, change? We addressed this issue by studying embodiment towards virtual avatars in 70 girls aged 10–17 years. We manipulated the synchrony between participants’ and avatars’ touch or movement, as well as the avatar visual shape or size relative to each participant’s body. A weaker avatar’s embodiment in case of mismatch between the body seen in virtual reality and the real body is indicative of a more robust bodily self-consciousness. In both the visuo-tactile and the visuo-motor experiments, asynchrony decreased ownership feeling to the same extent for all participants, while the effect of asynchrony on agency feeling increased with age. In the visuo-tactile experiment, incongruence in visual appearance did not affect agency feeling but impacted ownership, especially in older teenage girls. These findings highlight the higher malleability of bodily self-consciousness at the beginning of adolescence and suggest some independence between body ownership and agency.

## Introduction

Adolescence is a period of significant changes in body shape and size. These changes can lead to temporary clumsiness^1^, which typically disappears with the updating of processes underlying body schema^2^. At the same time, the neural networks that support perception–action coupling are still maturing, involving change in structural and functional connectivity between primary sensory areas (primary somatosensory and visual cortices) and areas important for multi-sensory integration, motor and higher-order functions (inferior parietal lobe, supplementary motor areas, primary motor cortex and inferior frontal gyrus)^3–5^. In parallel, adolescence is also  a period of development of self perception and of relationships with others^6^. For example, it is a period of improvement in self-concept clarity^7,8^. Brain areas involved in self-reflection (medial prefrontal cortex and medial posterior parietal cortex) are also undergoing maturation, in particular in their connectivity with areas involved in social processing (dorsomedial prefrontal cortex, temporoparietal junction, and posterior superior temporal sulcus)^9^. Sensorimotor and self-perception features are known to contribute, in adults, to bodily self-consciousness^10,11^ which can be defined as the implicit and pre-reflexive experience of being an embodied self^12,13^. In other words, bodily self-consciousness relates to the experience we have of being a ‘real me’ that “resides” in “my” body and is the subject (or the “I”) of our experiences, actions and thoughts^12^. Bodily self-consciousness is thus likely to be special and changing during adolescence. Yet, empirical studies of adolescent bodily self-consciousness are scarce.

One way to assess bodily self-consciousness, and the robustness of own-body representation upon which it relies, is to use embodiment illusions. When presented with an avatar in first person perspective via a virtual reality device while experiencing a tactile or motor stimulation matched to that of the avatar, most observers embody this avatar. More precisely, they have the feeling that the seen body is their own (ownership), that the felt touch is caused by the stimulus applied on the seen body (referral of touch), and that the seen action is generated by themselves (agency)^14^. These three feelings are subcomponents of bodily self-consciousness. When presenting an avatar in third-person perspective, the experimenter can also manipulate the participant’s sense of self-location and first-person perspective, which are considered to be other components of bodily self consciousness^12^. The embodiment experience is diminished or abolished when (i) there is a delay (asynchrony) between the tactile stimulation or movement of the avatar and the stimulation or movement of the participant’s body or when (ii) there is a mismatch between the avatar visual appearance and the physical appearance of the participant^15,16^. The strength of embodiment illusion and its modulation varies between individuals, however. For instance, a low clarity of self-concept^17^ is associated with increased embodiment of a rubber hand and less vulnerability to mismatch. Embodiment illusions can thus be used to assess the robustness *vs* the malleability of bodily self-consciousness: a reduction of the illusion by a tactile, motor, or visual mismatch indicates a robust bodily self-consciousness; and, conversely, an immunity of the illusion to a mismatch indicates a more malleable bodily self-consciousness. Considering that each sort of mismatch may have a different influence, manipulating the nature of the mismatch can inform on general or specific features of bodily self-consciousness. Also, different measures of embodiment can be differently affected by experimental manipulations^18^. For instance, Dupraz et al.^19^ showed that while self-reported ownership and agency over an avatar seen from a first-person perspective decreased when the subject’s and the avatar’s arm movements were desynchronized, the kinesthetic illusion, provoked by the movement of the avatar after the embodiment induction phase, was not affected by the asynchrony. This led the authors to conclude that an avatar can be treated as one’s own body, in terms of sensorimotor integration, even if it is not consciously perceived as such. Physiological measures can also inform on the arousing and affective effects of illusory embodiment. Bergstom et al.^20^ reported that heart rate variability was associated with the strength of ownership feeling, during a full-body ownership illusion (although Critchley et al.^21^ reported no such effect when using a more comprehensive Bayes analysis). D’Alonso et al.^22^ reported a correlation between skin conductance and measures of the rubber-hand illusion, although this correlation faded when the experiment was repeated. Therefore, combining these different measures can provide complementary information and thereby a global, although sometimes complex, picture of the malleability of bodily self-consciousness.

Previous research has shown that children as young as 4 can experience an embodiment illusion over an artificial limb, face or body^23,24^. Using a full-body illusion with a first-person perspective, Keenaghan et al.^25^ reported that in 5-years-olds, subjective ratings of ownership and agency over the avatar were not affected by the visuo-motor asynchrony nor by the size of the avatar, indicating a malleable bodily self-consciousness. Using the same experimental paradigm in 8–12 year-olds, Weijs et al.^26^ reported that an asynchrony in the avatar movement or an unrealistic avatar shape decreased the ownership feeling over the avatar in the same way as it did in adults. In contrast, the feeling of agency decreased less in children than in adults. Cowie et al.^27^ quantified embodiment objectively using drift in perceived self-location during a full-body illusion; they showed a greater drift in the synchronous condition than in the asynchronous condition for adults, but not for children aged 6 to 11 years. These results suggest that different subcomponents of bodily self-consciousness may follow different developmental trajectories. Also, the illusion appears quicker in adolescents than in adults^28^, implying that adolescents require less stimulation to experience the illusion. In a cross-sectional sample of 5- to 17- year-olds, embodiment of an adult-size rubber hand did not change with age, while embodiment of a small rubber hand decreased with age^29^, indicating that younger children were less impacted by a mismatch in hand size than adolescents, and therefore suggesting that bodily self-consciousness is less robust in children and become more robust through adolescence. How exactly the robustness/malleability of distinct aspects of bodily self-consciousness changes during the course of adolescence remains to be established, however.

The current study investigated responses to a full-body illusion in adolescents aged 10–17 years. We chose 10 as the lower age limit of our sample because it corresponds roughly to the beginning of puberty in girls. Many family and social changes occur after the age of 17, especially after high school is completed, therefore we set the upper limit at 17 to minimize confounding factors. As adolescent development timeline differs between sexes, we chose to focus only on girls. We tested bodily self-consciousness malleability with two experimental manipulations: (1) introducing a delay, i.e. an asynchrony between the tactile stimulation or movement of the avatar and the tactile stimulation or movement of the participant’s body, and (2) modulating the avatar visual appearance. For the latter, we used two orthogonal transformations: an avatar with a larger or smaller body mass index or an avatar with no (Child-like morphology) or full secondary sexual features (Adult-like hip-to-waist ratio and breast). We measured the impact of these manipulations on three components of bodily self-consciousness, namely ownership, referral-of-touch, and agency subjective feeling. We also recorded two kinds of objective indexes of embodiment: 1. a novel measure of implicit body perception; and 2. heart rate and skin conductance. It is indeed possible that embodying an avatar with distorted appearance relative to one’s own appearance, induces an implicit discomfort and thereby an autonomic response^20^. Also, previous developmental studies implementing full-body illusions have used either visuo-tactile^27^ or visuo-motor induction^26^. Here we chose to test the two types in the same participants, to determine whether visuo-tactile and visuo-motor modalities differently impact embodiment in adolescence.

We hypothesized that bodily self-consciousness is more malleable in early and mid-adolescence, when individuals are adapting to physical changes in size and appearance, to psycho-social changes related to the importance of body image, and to physiological changes related to sexual maturation. This should be reflected in less impact of avatar asynchrony and visual appearance than later in adolescence. This would be consistent with the idea that both multisensory integration and visual determinants of bodily self-consciousness become more robust during adolescence. As adolescent development is non-linear, we also considered that these effects could change non-linearly as a function of age. Lastly, in the case of avatar appearance manipulation, we expected a modification of implicit body-size estimation as well as of physiological markers of emotion and stress, and assessed in an exploratory way whether this would change with age.

## Material and methods

The study was approved by the ethics committee of Aix Marseille Université and all research was performed in accordance with relevant guidelines/regulations, in line with the declaration of Helsinki. This study was not preregistered. Upon publication data will be made available on OSF.

### Participants

The study involved two visits to the laboratory, a week apart. Seventy-eight girls aged 10–17 years enrolled in the study. No prior data was available from the literature to estimate effect sizes rigorously, so the sample size was determined in the present study based on previous developmental studies looking at similar effects (78 participants in Cowie et al.^27^ and 65 in Weijs et al.^26^). Four participants did not show up for the second session, and data from four others were discarded because of obvious disinterest or technical issues. The final sample consisted of 70 participants with a mean (± SD) age of 13.44 ± 2.20 years, and a mean BMI of 19.88 ± 3.21 (min. 14, max 27). Inclusion criteria were: identifying as a girl, being between 10 and 17 years of age, having no current, nor history of, neurological illness, and no current physical condition known to influence body weight and/or size (for example, pregnancy). None of the participants had extensive experience with virtual reality. Data on race/ethnicity could not be collected, due to legislation. Participants were recruited through convenience and steps in different local schools. All legal representatives and participants signed an informed consent form. Participants received 35 euros upon completing the two visits.

### Pre-experimental session

**Figure 1:**
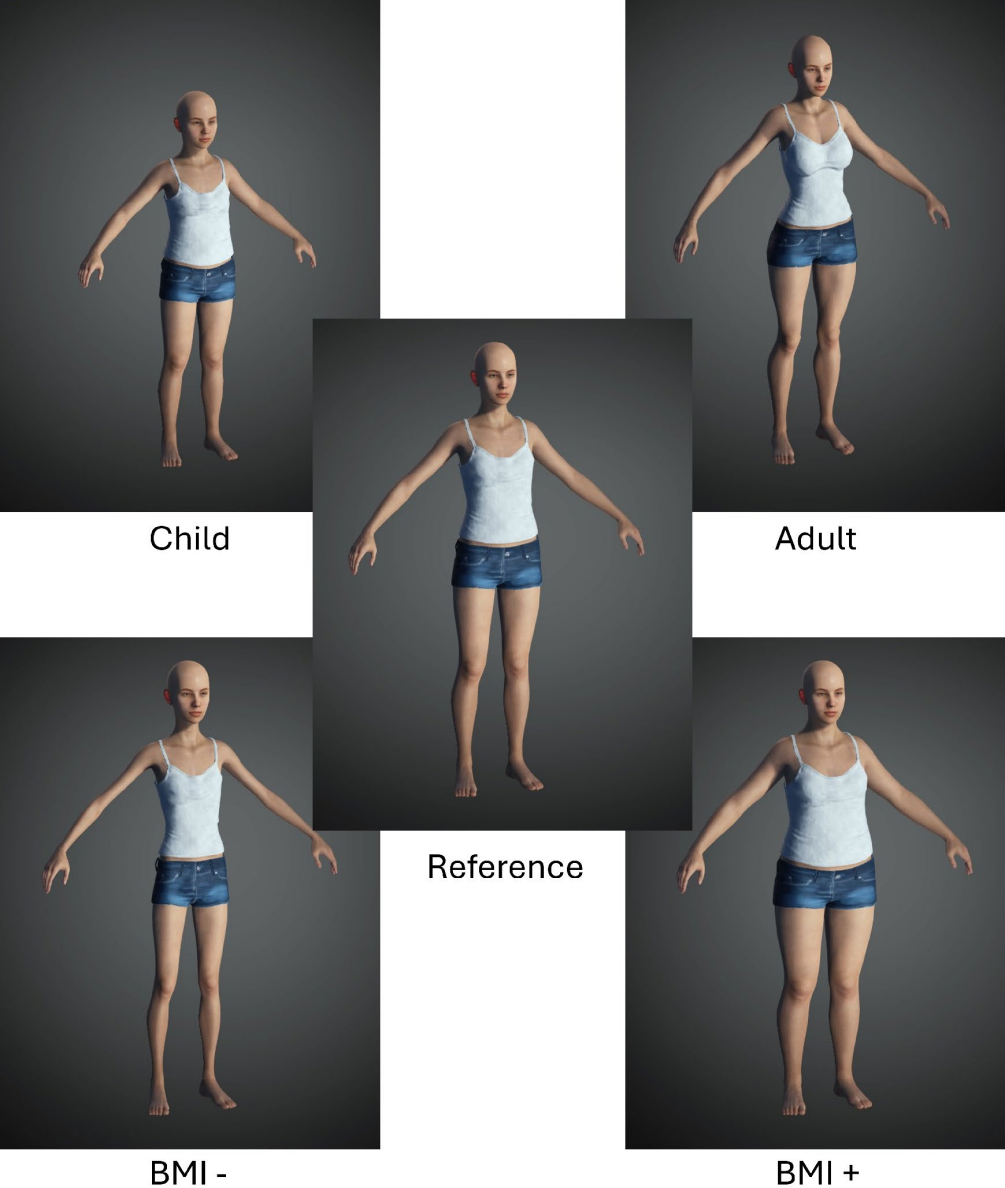
Example of five avatars made with the software Character creator and personalized according to the participant’s body measures and pictures: one avatar reproduces the participant’s real body (Reference), one without puberty sign (Child-like), one with puberty signs (Adult-like), one with a lower Body-Mass Index (BMI−), and one with a higher BMI (BMI +).

A pre-experimental session aimed at collecting demographic information and taking quality photographs in order to create a personalized avatar for each participant. A female experimenter took photos of each participant’s body standing (front, back, and profile view) using a CANON EOS 600 camera mounted on a tripod. We used the photos to create, for each participant, five avatars (Fig. 1) using Character Creator v 3.43 (Reallusion Inc). The first avatar, “Reference Avatar” matched the participant’s body in size, height and shape. From this, we created four additional avatars. The “BMI +” and “BMI−” avatar depicted a 15% gain or loss, respectively, of body mass index compared to the Reference avatar. The “Child-like” avatar showed no sign of pubertal development, i.e. no chest and hip development and high sitting height ratio (a larger sitting height ratio indicates relatively short legs for total stature; it decreases from childhood to end of puberty^30^. The “Adult-like” avatar depicted a woman after puberty (developed chest and hip and lower sitting height ratio). As the estimation of future height is difficult, and to some extent past height too, we used the same prototypical height for all participants for “Child-like” and “Adult-like” avatar which was, respectively, of 130 cm and 180 cm (the minimum height in our sample was 135 cm and the maximum 175 cm, mean 159 ± 9 cm). We imported these avatars into Unreal Studio (Unreal Engine) for the virtual environment used in the experimental session.

### Experimental session

**Figure 2:**
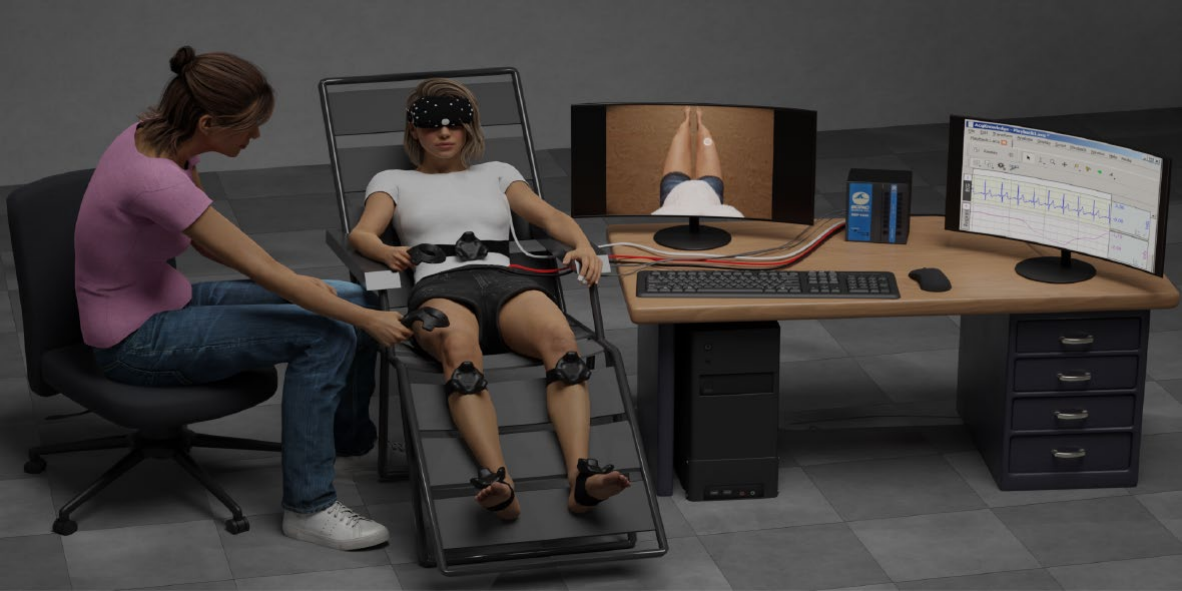
Experimental set-up (for Experiment 1). Participants were on a recliner chair and were immersed in a virtual environment through a head-mounted display. They saw the avatar from a first-person perspective. A female experimenter could use a VR controller to gently stroke the participants’ right thigh, and the participant saw in the virtual environment (shown on the central monitor) a grey ball that stroked the avatar’s right thigh. Electrocardiogram (ECG) and electrodermal activity (EDA) were recorded with chest and palmar electrodes. For the illustrative purpose of this figure, and to preserve the participants and experimenter’s anonymity, we made virtual characters using the database of Character Creator (Reallusion, https://www.reallusion.com/character-creator/).

The experimental session lasted approximately 1.5 h. Participants were seated in a recliner chair and were immersed in a virtual environment through a head-mounted display (HMD; HTC Vive, HTC Corporation) (Fig. 2). To record participants’ posture and movements, and synchronize them with those of the avatar, five motion trackers were placed on the belly, knees, and feet. Head movements were tracked to adjust visual perspective in real time. Participants held a VR controller in their right hand. The virtual environment depicted a neutral room in which the light could be turned off and a screen could appear to present questions. We conducted two experiments with a 10-min break in between. Embodiment illusions were induced through visuo-tactile stimulation in Experiment 1, and through visuo-motor stimulation in Experiment 2. In both experiments, we investigated the impact of two kinds of mismatch on embodiment: Asynchrony and Avatar appearance. To assess the impact of asynchrony, we manipulated the delay between tactile stimulation and visual display of touch on the virtual body (Experiment 1) and between the participant’s movements and the visual display of the avatar’s movements (Experiment 2). To assess the impact of avatar appearance, we manipulated the avatar’s shape and size using the five custom avatars described above (Reference, Child-like, Adult-like, BMI +, BMI −). Each experiment consisted of 10 trials (2 synchrony conditions * 5 avatar conditions) presented in counterbalanced order. The rest period between trials was approximately one minute long, but could be longer if participants so required.

#### Experiment 1: visuo-tactile induction of embodiment

Before the start of each trial, the light was turned off in the virtual environment. A calibration was performed to ensure a precise overlap between the participant’s posture and the avatar’s posture and to enable congruent visuo-tactile stimulation. Then the following three phases were presented:

- **Embodiment phase – First explicit report of embodiment.** The light was turned on in the virtual scene. The participant saw, from a first-person perspective, a virtual avatar whose posture matched her own body posture. A female experimenter used a VR controller to gently stroke the participants’ right thigh along an 8-cm line. The controller’s movement was depicted in the virtual environment as a grey ball that continuously stroked the avatar’s right thigh in the same body space and time in the synchronous condition. In the asynchronous condition, the grey ball reproduced the movement of the controller on the avatar’s leg in the same location but with a 500 ms delay. This delay was chosen based on a previous experiment showing that it reduced embodiment^31^. The experimenter ensured that the participant looked at the avatar by continuously checking the PC monitor that showed the participant’s field of view in real time. Participants were instructed to press a button on the VR controller when they agreed with the statement “I feel like the ball I see is touching my own body” (as in^32^). Whether or not participants pressed the button, the embodiment period lasted 75 seconds. We recorded electrocardiogram (ECG) and electrodermal activity (EDA) throughout.
- **Ball contact estimation task—Implicit measure of embodiment.** We developed a ball contact estimation task, inspired by Nico et al.’s task^33^ used to assess implicitly the subjective body size estimation. When the visuo-tactile stimulation stopped, a red ball appeared 60 cm in front of the participant. This ball moved at a constant speed of 18 cm/sec towards the participant’s belly. When it reached a distance of 20 cm from the participant’s belly, the environment became dark, leaving only the moving ball visible. Participants were instructed to press the VR controller button when they believed the ball would make contact with their physical belly. We recorded the coordinates of the ball and the participant’s physical belly at the time of button press.

Completing the ball contact estimation task took approximately 5 seconds.

**Questionnaire—Second explicit measure of embodiment.** Participants were asked to rate their subjective experience during the visuo-tactile stimulation in Phase 1 on a seven-item questionnaire (see Table 1) presented in the virtual environment. Three questions pertained to ownership (the feeling of the virtual body being one’s own), three others pertained to referral of touch (the feeling of directly being touched by the seen ball) and one to the feeling of nausea. Using the VR controller, participants answered the questions on a visual analog scale ranging from 0 (completely disagree) to 100 (completely agree). The order of the questions was counter-balanced across trials and participants. Completing the questionnaire took about one minute.

**Table 1:** Questions for explicit embodiment measures.

Questions
I felt like I was seeing my own body *(ownership)*
I felt like the virtual body belonged to me *(ownership)*
I felt like I was in the virtual body *(ownership)*
The ball I saw was touching me *(reference of touch)*
The touch I felt was caused by the ball I saw *(reference of touch)*
I felt the touch where the virtual body was touched *(reference of touch)*
The virtual body could move as I wanted* (agency)*
I could control the movements of the virtual body *(agency)*
I could move the legs of the virtual body as I wanted *(agency)*
I felt nauseous

Before Experiment 1 started, participants were familiarized with the instructions and handling of the VR controller. They were asked not to move and to look towards their abdomen to prevent unnecessary head movement and to ensure constant visibility of the avatar’s belly and legs. The instructions were repeated just before each trial.

#### Experiment 2: visuo-motor induction of embodiment

As in Experiment 1, a trial was composed of the following three phases:

- **Embodiment phase.** The light was turned on in the virtual scene and participants saw a virtual avatar from a first-person perspective. Participants were instructed to start a set of feet and knees flexions and extensions that had been previously demonstrated to them by the experimenter. The avatar’s legs moved synchronously with the participants’ legs in the synchronous condition, and with a 500 ms delay in the asynchronous condition. Participants were instructed to indicate, by pressing a button on the VR controller, when they agreed with the following statement “I feel like I see my own body moving”. The embodiment period lasted 75 seconds.
- **Ball contact estimation task.** Same as in Experiment 1.
- **Questionnaire.** Three questions pertained to ownership, three pertained to agency (the sense of being in control of the virtual body) and one to the feeling of nausea (Table 1).

Before Experiment 2 started, participants were familiarized with the leg movements and the instructions for the three phases were repeated.

### Behavioral data processing

We analyzed the following variables to characterize embodiment:

- **Explicit reports:**

- *Occurrence*: a binary measure based on whether participants pressed (1), or not (0), the VR controller during the 75 seconds of embodiment phase to indicate that they felt that their own body was being touched or moved.
- *Onset time*: For trials in which the illusion occurred, the time (in sec) between the beginning of the trial and the button press.
- *Subjective report of ownership and referral of touch (Exp 1) or agency (Exp 2)*: Ratings on visual analog scales for the three corresponding questions (see Table 1).

- **Implicit measure:**

- *Ball-to-Belly Distance*: the distance (in cm) between the perceived belly boundary (i.e., coordinates of the ball when participants indicated it touched their belly) and the real belly boundary. An increased absolute distance when the avatar’s trunk was larger (Adult-like and BMI + conditions) or smaller (Child-like and BMI− conditions) than the trunk of the Reference avatar would indicate embodiment of these avatars, or in other words, an incorporation of the avatar’s features into the participant’s own body representation.

We had some missing data, due to technical issues or participants reporting a mistake, in Experiment 1: 2.1% for illusion occurrence and onset time, 0.7% for subjective reports, 5.0% for ball-to-belly distance, and in Experiment 2: 4.0% for occurrence and onset time, 2.5% for subjective reports, 5.7% for ball-to-belly distance. We converted raw onset times into logarithms prior to statistical analysis to account for the fact than a 1 s delay in onset time at the beginning of the stimulation has more importance that a 1 s delay after a longer stimulation period. This transformation also produced a normal distribution. To account for idiosyncratic use of the visual analog scales, we transformed the ratings into Z-scores at the participant level: for each participant, we computed the average and standard deviation across responses on all questions from both experiments and then substracted the average from each score and divided this by the standard deviation. This ipsatization process neutralizes the effect of scales usage and also produces a distribution of scores that can be analysed with parametric tests, while minimizing the effect of outlier values^34,35^ (see supplementary material S1 for analyses of raw data).

### Physiological data recording and preprocessing

We determined how our experimental conditions impacted the participants’ heart rate variability (HRV) and skin conductance responses (SCR), which are markers of, respectively, mental stress^36,37^ and arousal induced by emotional and cognitive load^38^. Both measures have been used in studies of illusion to provide an implicit assessment of embodiment complementary to explicit questionnaires^20,22^. We recorded electrocardiography (ECG) and electrodermal activity (EDA) during the 75 seconds of stimulation for each trial. We used a BIOPAC MP160 (BIOPAC Inc., Goleta, CA, USA) system with AcqKnowledge 5.0 software (BIOPAC Inc., Goleta, CA, USA). We preprocessed data using the Python package NeuroKit2^39^ (see supplementary material S1).

For the ECG, R-peaks were identified automatically from the filtered data using the NeuroKit2 algorithm. From each 75-s period of stimulation, we derived three HRV measures: the root-mean-square of successive differences in R-R intervals (**HRV_RMSSD** in ms), the absolute power of the high-frequency band (**HRV_HF** in ms2) and the Poincaré plot standard deviation along the line of identity (**HRV_SD2** in ms). HRV_RMSSD is one of the most commonly reported HRV indices, but it does not distinguish the respective contributions of sympathetic and parasympathetic regulatory mechanisms of HRV. In contrast, HRV_HF is suited to assess the parasympathetic component of HRV^36^. HRV_SD2 is a measure introduced more recently derived from non-linear methods; it is believed to reflect sympathetic activity^36,40^. HRV_RMSSD has been shown to decrease during acute stress, while HRV_SD2 and HRV_HF increase. These three measures are valid for capturing short-term HRV features within a 75-s epoch^41^. We had some missing data due to technical problems during acquisition: 10.8% (ranging from 10.0 to 12.9% across conditions) in Experiment 1 and 11.8% for (ranging from 11.4 to 12.9%) in Experiment 2.

For the EDA, skin-conductance responses (SCRs) were identified when peaks in the tonic skin conductance signal exceeded 0.01 $\upmu$s, with a rejection threshold set at 10% of the participant’s largest peak to exclude most of the noise. The amplitude of each SCR was determined as the peak value relative to the value at the onset of the initial deflection of this response. Two measures were extracted for subsequent statistical analysis: (a) the maximum amplitude of the skin conductance responses occurring within 15 s after the start of each trial, reflecting the sympathetic response to the onset of the avatar presentation (**SCR_Amplitude** in $\upmu$s) and (b) the number of SCRs occurring during the whole stimulation phase (**SCR_Number** in unit), reflecting the sustained sympathetic activity during the duration of the trial. These measures of SCR have been shown to increase with the emotional load^38^. We encountered 28.8% missing data across both experiments (ranging from 28.6 to 30.0% across participants) due to technical issues or participants not exhibiting a response in EDA, which is consistent with the typical prevalence of 10–25% EDA hypo-responders in the population^42^.

### Statistical analyses

All statistical analyses were performed using R software (version 4.2) using the RStudio interface. We analyzed each experiment separately and set significance for hypotheses and models testing at p < 0.05. We fitted a logistic model (estimated via Maximum Likelihood) for Occurrence, and linear mixed models (estimated via restricted Maximum Likelihood) for the other measures, using *‘lme4′*^43^. All the models included subject as random effect (~ 1 | suj) and, as fixed effects, Asynchrony (Synchronous or Asynchronous visuo-tactile or visuo-motor stimulation), Avatar (Reference, BMI+, BMI−, Child-like, Adult-like), Age (in months), as well as their interactions (Measure ~ Age + Avatar + Asynchrony + Age:Asynch + Age:Avatar + Asynch:Avatar + Age:Asynch:Avatar + (~ 1 | suj)). To explore potential non-linearity in development, we repeated the procedures with quadratic and cubic terms for the Age covariate and compared the resulting models to the model with a linear term with likelihood ratio tests^44^ using the *‘lmtest’* package for logistic, and Wald F-test with Kenward-Roger approximation for degrees of freedom (as recommended by Spieler^45^) using the *‘lmerTest’* package^46^ for linear models. Models’ diagnostics were performed visually (homoscedasticity and normality of residuals plots inspection).

When the main effect Avatar was significant, we conducted posthoc pairwise comparisons between each modified avatar and the Reference avatar, as well as between BMI + avatar and BMI− avatar and between Adult-like avatar and Child-like avatar, adjusting p-values for 6 comparisons using false discovery rate (FDR). For logistic models, we used Wald’s Z-tests and for linear and polynomial models, we used Wald’s T-tests. When the interaction between Age and Asynchrony was significant, we first analyzed the direction of the effect of Age on the difference between synchronous and asynchronous conditions using F-tests (logistic models) or Wald T-tests (linear and polynomial models). Then, we investigated the effect of Age on the synchronous and asynchronous conditions separately using separate mixed models. When the interaction between Age and Avatar was significant, we examined the effect of Age on each avatar separately, with five mixed models (one for each level of Avatar). Additionally, we performed pairwise comparisons between the effect of age on the Reference avatar and the effect of age on each other avatar, as well as between the effect of age on the Adult-like and on the Child-like avatar. We used Wald’s T-test with p-values FDR corrected for 5 comparisons. Lastly, in cases when the interaction with Age was significant, we further examined non-linear effects by incorporating the second or third power of age into the analysis. When the model with such a polynomial function explained significantly more variance than the model with linear age interaction effect, we determined the predicted age corresponding to the inflexion point of this function.

## Results

Please note that additional quantitative details for the statistical results, including coefficients and their confidence intervals and models’ fit, are to be found in supplementary information S1.

### Experiment 1: visuo-tactile induction of embodiment

In this experiment, we stroked the participants’ right thigh while a ball simultaneously stroked the avatar’s leg to induce embodiment towards the avatar. We investigated how embodiment was influenced by a delay between the participant’s and avatar’s touch, as well as by the mismatch between the participant’s and avatar’s body appearance. We did not observe any significant Asynchrony × Avatar, nor any Age × Asynchrony × Avatar interaction (see supplementary material S1). We therefore report results based on statistical models without these interaction terms. Statistics for main and interaction effects are reported in Table 2. Models with quadratic and cubic age term in interaction with synchrony were better than a linear term to predict onset time (X2(2) = 10.04, p = 0.007) and referral of touch (X2(4) = 9.81, p = 0.044), respectively.

**Table 2:** Main and interaction effects on the different measures in the Visuo-Tactile Experiment.

Measures	Asynchrony	Avatar	Age × Asynchrony	Age × Avatar
Occurrence	**X**^**2**^**(1) = 193.42**	X^2^(4) = 0.66	X^2^(1) = 0.81	X^2^(4) = 3.52
	**p < 0.001**	p = 0.956	p = 0.368	p = 0.474
Onset time	**F(1,481) = 122.19**	F(4,468) = 0.66	**F(2,479) = 17.78**	F(4,468) = 0.54
	**p < 0.001**	p = 0.622	**p < 0.001**	p = 0.706
Ownership	**F(1,615) = 69.88**	**F(4,615) = 6.15**	F(1,615) = 0.06	**F(4,616) = 3.48**
	**p < 0.001**	**p < 0.001**	p = 0.812	**p = 0.008**
Ref touch	**F(1,613) = 242.81**	F(4,613) = 0.38	**F(3,613) = 14.67**	F(4,614) = 1.6
	**p < 0.001**	p = 0.825	**p < 0.001**	p = 0.174
Ball-Belly dist	F(1,587) = 0.13	**F(4,587) = 13.12**	F(1,587) = 0.02	F(4,587) = 1.71
	p = 0.72	**p < 0.001**	p = 0.902	p = 0.146
HRV RMSSD	F(1,551) = 2.73	F(4,551) = 1.18	F(1,551) = 2.37	F(4,551) = 0.66
	p = 0.099	p = 0.318	p = 0.124	p = 0.62
HRV HF	F(1,551) = 0.22	F(4,551) = 0.67	F(1,551) = 0.74	F(4,551) = 0.74
	p = 0.639	p = 0.611	p = 0.391	p = 0.566
HRV SD2	F(1,551) = 1.08	F(4,551) = 0.3	F(1,551) = 1.15	F(4,551) = 0.23
	p = 0.299	p = 0.877	p = 0.283	p = 0.923
SCR amplitude	F(1,438) = 0	F(4,438) = 0.56	F(1,438) = 0.57	F(4,438) = 0.6
	p = 0.989	p = 0.693	p = 0.451	p = 0.66
SCR number	F(1,438) = 0.05	F(4,438) = 1.84	**F(1,438) = 5.43**	F(4,438) = 0.65
	p = 0.819	p = 0.119	**p = 0.02**	p = 0.629

Significant effects are highlighted in bold. Values for the interaction Age x Synchrony for onset time and referral of touch ratings correspond to models with quadratic and cubic age terms.

#### Effect of asynchrony

##### Main effect

**Figure 3:**
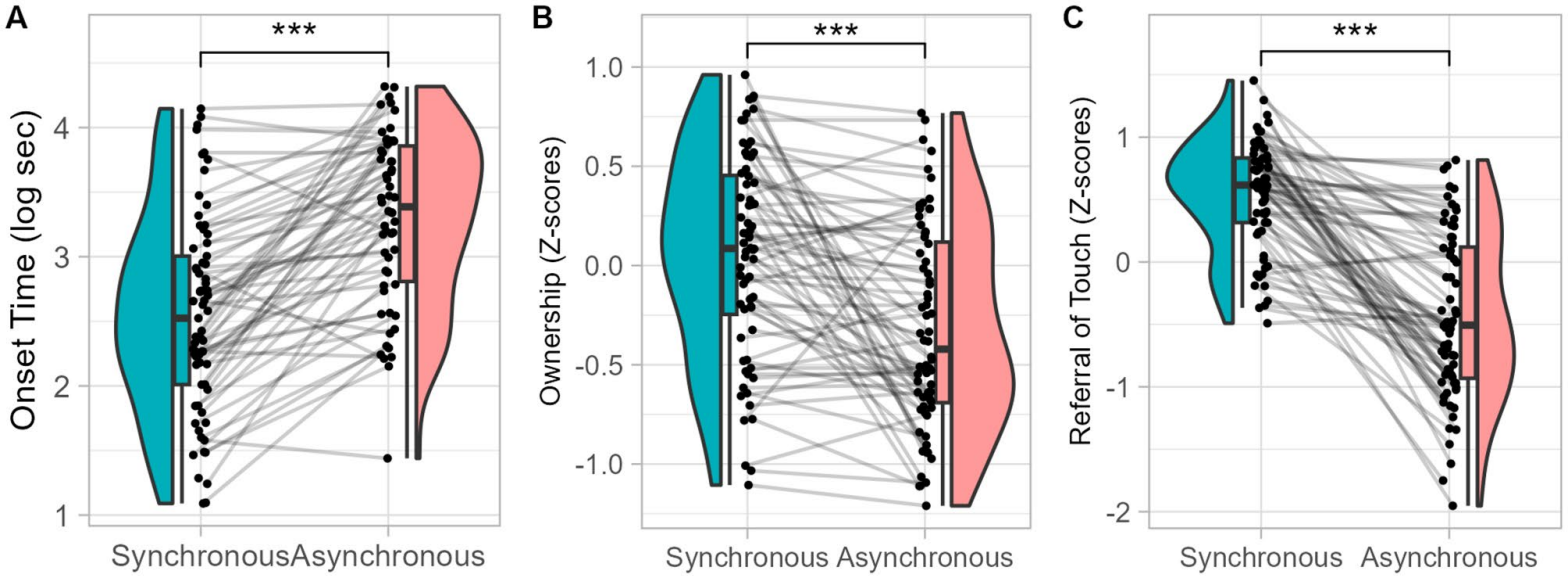
Effect of Asynchrony on explicit measures in Experiment 1 (visuo-tactile induction). Distribution of onset time (**A**), ownership (**B**) and referral of touch (**C**) in synchronous (blue) and asynchronous (red) conditions. ***p < .001.

We observed a main effect of Asynchrony on all our explicit measures of the illusion (Fig. 3). In asynchronous compared to synchronous condition, the probability of illusion occurrence was lower (96.78% vs 62.68), onset time was longer and ownership ratings and referral of touch ratings were lower (p < 0.001). We did not observe any significant effect of Asynchrony on the implicit nor on the physiological measures.

##### Interaction Age × Asynchrony

**Figure 4:**
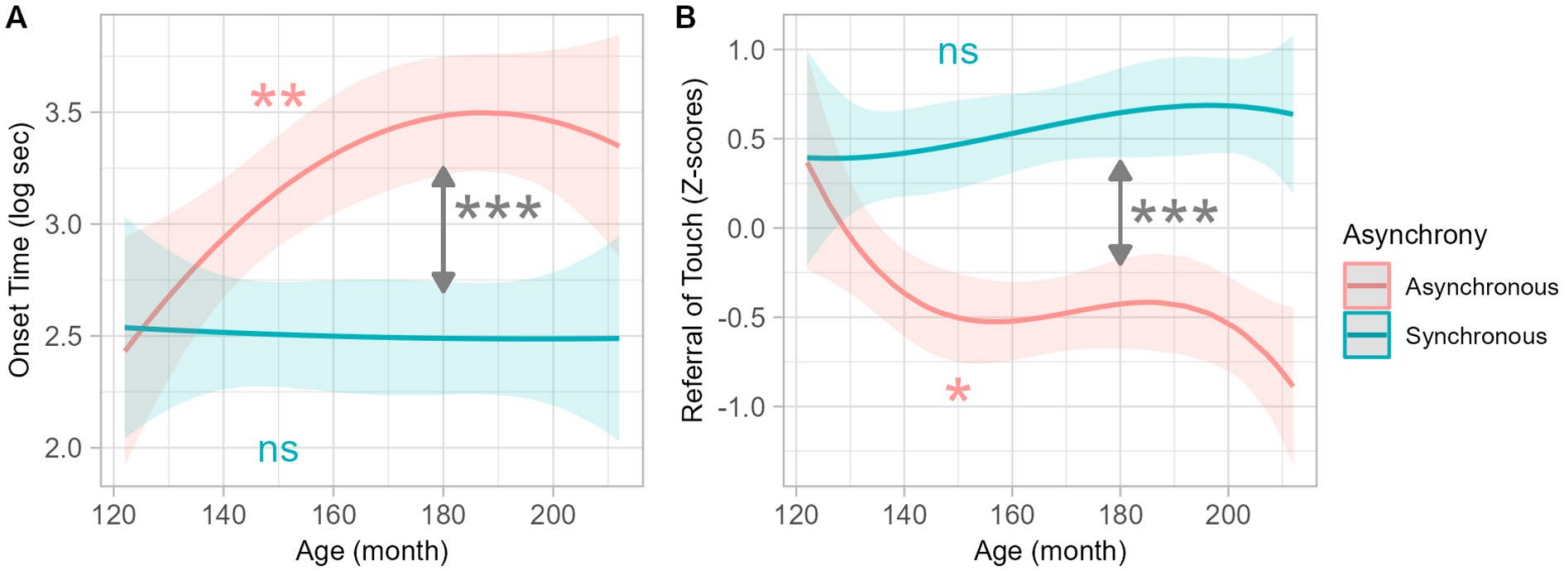
Interaction between Age and Asynchrony on explicit measures in Experiment 1 (visuo-tactile). Lines represent the polynomial fit of the data for onset time (**A**) and referral of touch (**B**) in synchronous (blue) and asynchronous (red), with the 95% confidence interval indicated by shaded areas (collapsed over the 5 avatar appearance conditions, as there is no interaction Avatar*Asynchrony). ***p < .001.

We observed an interaction between Age and Asynchrony on onset time, referral of touch ratings (Fig. 4), and the number of peaks in skin conductance, but not on the other measures.

For onset time, the Age × Asynchrony interaction was better described by a quadratic relation. Post-hoc analyses showed no significant change with age in the synchronous condition (p = 0.97), while in the asynchronous condition onset time increased non-linearly with age (p = 0.003). Analysis of the derivative of this function showed an inflection point at 187 months (15.5 years), after which there was no noticeable change with age. For referral of touch ratings, the Age × Asynchrony interaction was better described by a cubic relation. Post-hoc analyses showed no significant effect of age in the synchronous condition (p = 0.33) but in the asynchronous condition, referral of touch ratings decreased non-linearly with age (p = 0.030). The derivative analysis revealed a steep decrease from 120 to 159 months (13.2 years), followed by a plateau until 183 months (15.3 years) before decreasing again. Post-hoc analyses on the number of peaks in skin conductance showed no significant significant effect of age in the synchronous condition (p = 0.74) nor in the asynchronous condition (p = 0.35).*p < .05;  **p < .01;  ***p < .001.

#### Effect of avatar

##### Main effect

For the explicit measures, manipulating the avatar’s shape impacted only the ratings of ownership feeling. We also observed a significant effect on our implicit measure, namely ball-to-belly distance. We conducted post-hoc analyses separately for the manipulation of maturational shape and for the manipulation of BMI.

**Maturational shape manipulation** Compared to the Reference avatar, the Adult-like avatar led to a greater ball-to-belly distance (− 1.33 cm vs + 1.31 cm, p = 0.021). The difference between Child-like and Reference avatar was not significant (p = 0.58) while the difference between Adult-like and Child-like avatar was significant (p = 0.006; + 1.31 cm vs − 0.27 cm) with ball-to-belly distance varying by 1.58 cm. The difference in ownership ratings was not significant when comparing Reference to Adult-like avatar (p = 0.209) and showed a trend for being greater for the Reference compared to the Child-like avatar (p = 0.056). The difference between Adult- and Child-like avatar was not significant (p = 0.402).

**Figure 5:**
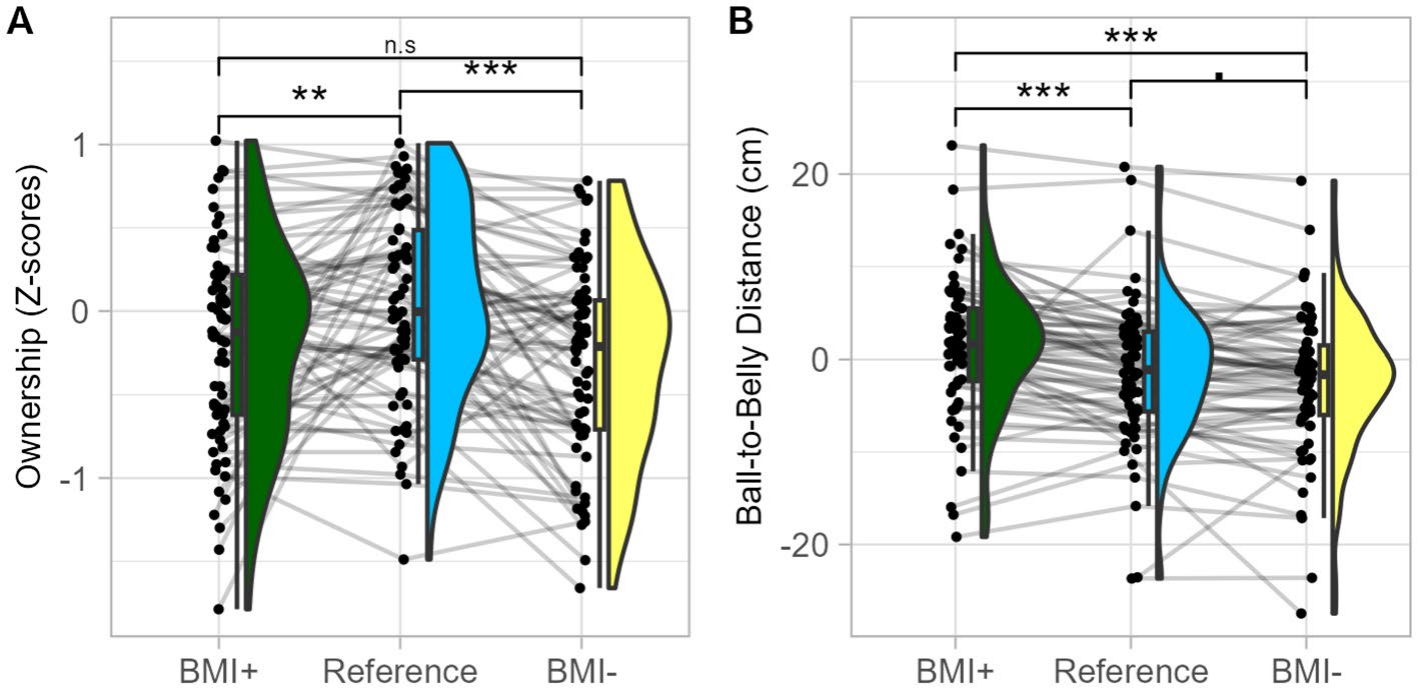
Effect of avatar’s body mass index manipulation in Experiment 1 (visuo-tactile). Distribution of ownership ratings (**A**) and ball-to-belly distance (**B**) in the condition of Avatar BMI + (green), Reference (blue) and BMI− (yellow). *p < .05; **p < .01; ***p < .001.

**BMI manipulation** Compared to the Reference avatar, ownership feeling decreased for the BMI + (p = 0.004) and BMI− (p < 0.001) avatar (Fig. 5A). The difference between BMI + and BMI− was not significant (p = 0.209). Compared to the Reference avatar, BMI + increased the ball-to-belly distance by 2.35 cm (p < 0.001) and BMI− tended to decrease it by − 0.99 cm (p = 0.068). The difference between BMI + and BMI− was significant (p < 0.001) (Fig. 5B).

##### Interaction Age × Avatar

We observed an interaction between Age and Avatar on ownership ratings, but not on the other explicit nor implicit and physiological measures.

**Figure 6:**
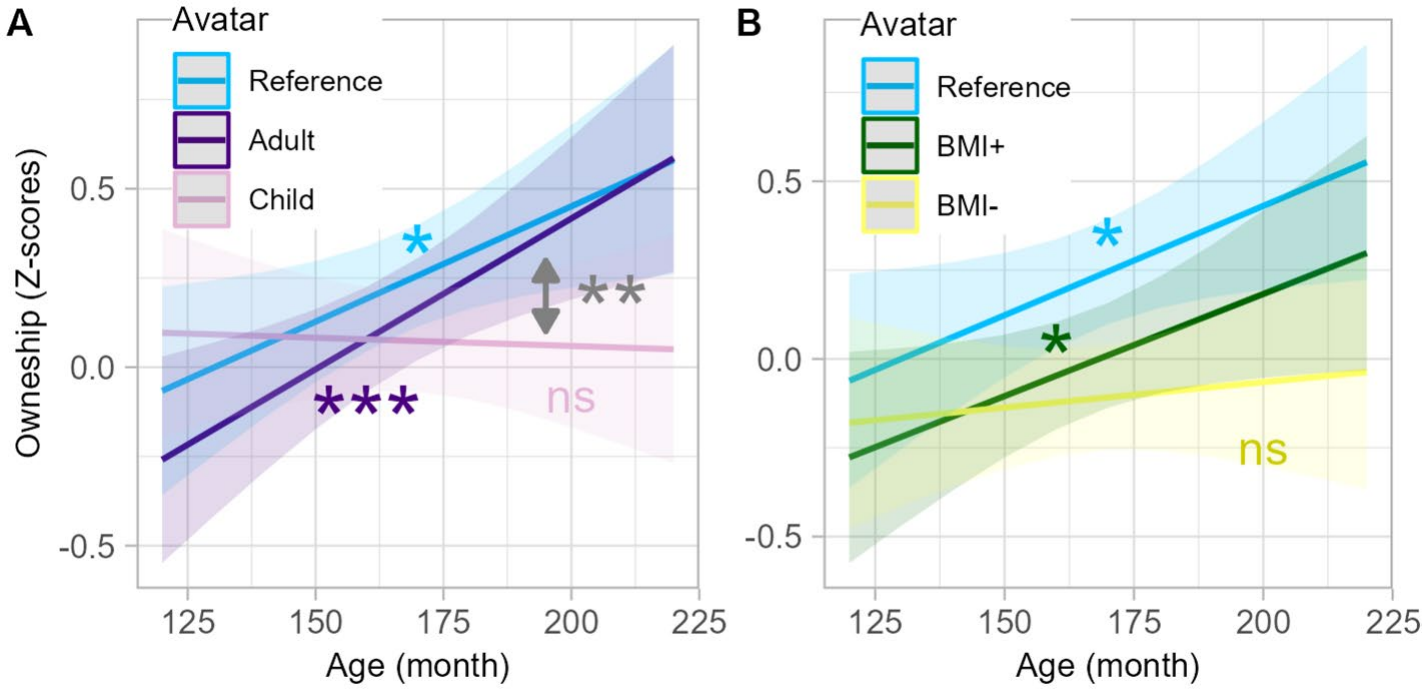
Interaction between Age and Avatar appearance on ownership in Experiment 1 (visuo-tactile). Lines represent the linear fit of the data as a function of age for avatars of different maturational shape (**A**) or body mass index (**B**), with the 95% confidence interval indicated by shaded areas. *p < .05; **p < .01; ***p < .001.

**Interaction age × maturational shape manipulation** Post-hoc analysis revealed that this interaction was driven by ownership feeling towards the Reference avatar (p = 0.018) and the Adult-like avatar (p = 0.001) increasing with the age of participants, while there was no significant age effect for ownership ratings of the Child-like avatar (p = 0.798). Pairwise comparisons (FDR corrected for 5 comparisons) showed that the difference of ownership ratings between the Child-like avatar and the Reference (p = 0.036) and the Adult-like avatar (p = 0.007) increased with the age of participants. There was no significant effect of age on the difference in ownership ratings between the Reference and the Adult-like avatar (p = 0.593) (Fig. 6A).

**Interaction Age × BMI manipulation** Ownership feeling towards the BMI + avatar increased with age (p = 0.036) while there was no significant age effect for the BMI− avatar (p = 0.622). Pairwise comparisons (FDR corrected for 5 comparisons) showed no significant effect of Age on the difference in ownership ratings between the Reference avatar and the BMI− avatar (p = 0.154) nor between the Reference avatar and the BMI + avatar (p = 0.886) (Fig. 6.B).

### Experiment 2 with visuo-motor induction of embodiment

In this experiment, we aimed at inducing embodiment by having participants perform simple movements while the avatar’s leg moved. We investigated how embodiment was affected by a delay between the participants’ movements and those of the avatar, and by manipulation of the avatar’s appearance. We did not observe any significant Asynchrony × Avatar, nor Age × Asynchrony × Avatar interaction (see supplementary material S1). Statistics for main and interaction effects are reported in Table 3. A model with a quadratic age term in Age × Avatar interaction predicted onset time better than the model with a linear term (p = 0.028). As for agency ratings, the model with a cubic age term described the Age × Asynchrony interaction better than the model with the linear age term (X2(4) = 20.89, p < 0.001).

**Table 3:** Main and interaction effects on the different measures in the Visuo-Motor Experiment (Exp2).

Measures	Asynchrony	Avatar	Age × Asynchrony	Age × Avatar
Occurrence	**X**^**2**^**(1) = 349.85**	X^2^(4) = 3.09	X^2^(1) = 1.05	X^2^(4) = 0.27
	**p < 0.001**	p = 0.543	p = 0.306	p = 0.992
Onset time	**F(1,322) = 49.1**	F(4,288) = 1.74	F(1,317) = 3.57	F(9,300) = 1.79
	**p < 0.001**	p = 0.141	p = 0.06	p = 0.069
Ownership	F(1,603) = 378.68	**F(4,604) = 6.23**	F(1,604) = 2.36	F(4,604) = 0.72
	p < 0.001	**p < 0.001**	p = 0.125	p = 0.576
Agency	**F(1,601) = 377.27**	F(4,602) = 0.96	**F(3,601) = 21.78**	F(4,602) = 0.42
	**p < 0.001**	p = 0.429	**p < 0.001**	p = 0.792
Ball-Belly dist	F(1,583) = 0.24	**F(4,583) = 11.49**	F(1,583) = 0.11	F(4,583) = 1.34
	p = 0.623	**p < 0.001**	p = 0.736	p = 0.252
HRV RMSSD	F(1,545) = 3.03	**F(4,545) = 2.48**	F(1,545) = 0.16	F(4,545) = 0.49
	p = 0.082	**p = 0.043**	p = 0.686	p = 0.742
HRV HF	F(1,545) = 2.38	**F(4,545) = 2.93**	F(1,545) = 0	F(4,545) = 0.74
	p = 0.124	**p = 0.021**	p = 0.995	p = 0.568
HRV SD2	**F(1,545) = 9.93**	F(4,545) = 1.26	F(1,545) = 1.95	F(4,545) = 1.29
	**p = 0.002**	p = 0.285	p = 0.163	p = 0.274
SCR amplitude	**F(1,438) = 4.39**	F(4,438) = 0.41	F(1,438) = 0.13	F(4,438) = 0.37
	**p = 0.037**	p = 0.799	p = 0.714	p = 0.828
SCR number	**F(1,438) = 12.88**	F(4,438) = 1.01	F(1,438) = 0.18	F(4,438) = 0.57
	**p < 0.001**	p = 0.403	p = 0.676	p = 0.686

Significant effects are highlighted in bold. Values for the interaction Age × Avatar for onset time and Age × Synchrony for agency rating correspond to models with quadratic and cubic age terms.

#### Effect of asynchrony

##### Main effect

**Figure 7:**
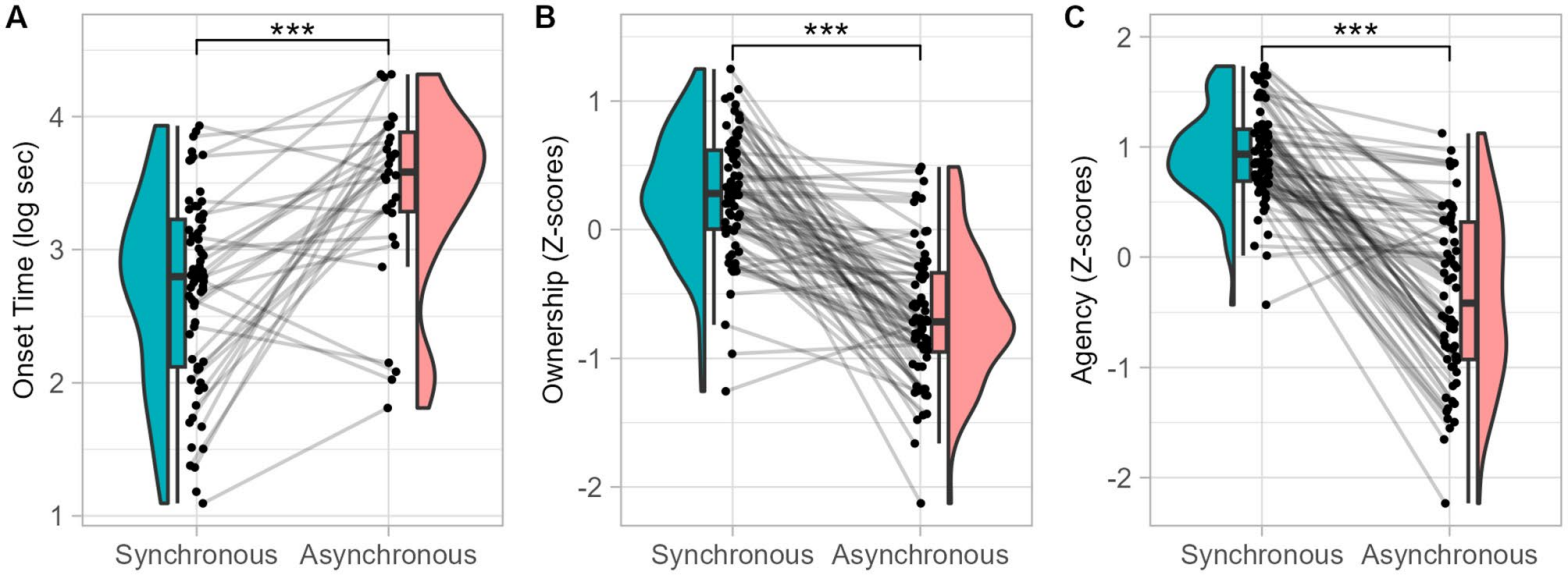
Effect of Asynchrony on explicit measures in Experiment 2 (visuo-motor). Distribution of onset time (**A**), ownership (**B**) and referral of touch (**C**) ratings in synchronous (blue) and asynchronous (red) conditions. ***p < .001.

As in Experiment 1, we observed a main effect of Asynchrony on all our explicit measures (Fig. 7) but not on the implicit measures. In the asynchronous compared to the synchronous condition, the probability of participants reporting illusion occurrence decreased (85.97% vs 20.47%, p < 0.001), the onset time increased and ownership and agency ratings decreased. Also, in the asynchronous condition, heart-rate variability non-linear component (SD2 index) increased (p = 0.002) and the number of peaks in skin conductance (p < 0.001) and their amplitude (p = 0.037) decreased.

##### *Interaction Age* × *Asynchrony*

**Figure 8:**
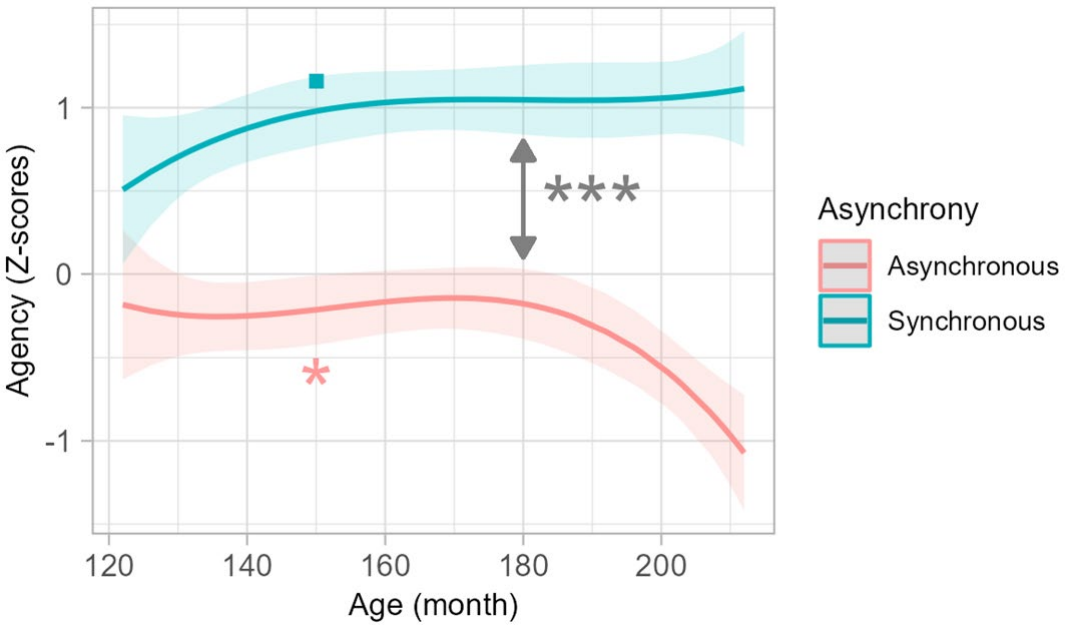
Interaction between Age and Asynchrony on explicit measures in Experiment 2 (visuo-motor). Lines represent polynomial fit of the data for agency ratings in synchronous (blue) and asynchronous (red), with the 95% confidence interval indicated by shaded areas. ***p < .001.

We observed an interaction between Asynchrony and Age on agency ratings (Fig. 8) but not on the other measures. The interaction was better described by a cubic relation: the difference between synchronous and asynchronous conditions increased non-linearly with age. Agency feeling increased slightly with age in the synchronous condition (p = 0.051), while it decreased markedly and non-linearly in the asynchronous condition (p = 0.037). Analyses of the derivative of this function showed a steep decrease from 120 to 137 months (11.5) years and then a plateau until 168 months (14 years) before decreasing again.

#### Effect of avatar

##### Main effect

For the explicit measures, manipulating the avatar’s shape significantly impacted only the ratings of ownership feeling. As in Experiment 1, we observed a significant effect on our implicit measure and on heart rate variability (HRV_HF and HRV_RMSSD).

**Maturational shape manipulation** When presented with an avatar with Child-like features, participants reported a weaker feeling of ownership than when presented with the Reference avatar (p = 0.023). Ownership ratings for the Adult-like avatar did not significantly differ from ownership ratings for the Reference (p = 0.104) nor Child-like avatar (p = 0.578). For the implicit measure of the illusion (ball-to-belly distance), post-hoc analyses did not reveal a significant difference between Reference and Adult-like (p = 0.135) nor Child-like (p = 0.962) avatar, neither was there any significant difference between Adult-like and Child-like avatar (p = 0.135). Likewise, for heart-rate variability, there were no significant difference between the Reference avatar and the Child-like (p = 0.155) nor the Adult-like (p = 0.175) avatar, nor between these two conditions (p = 0.81).

**Figure 9:**
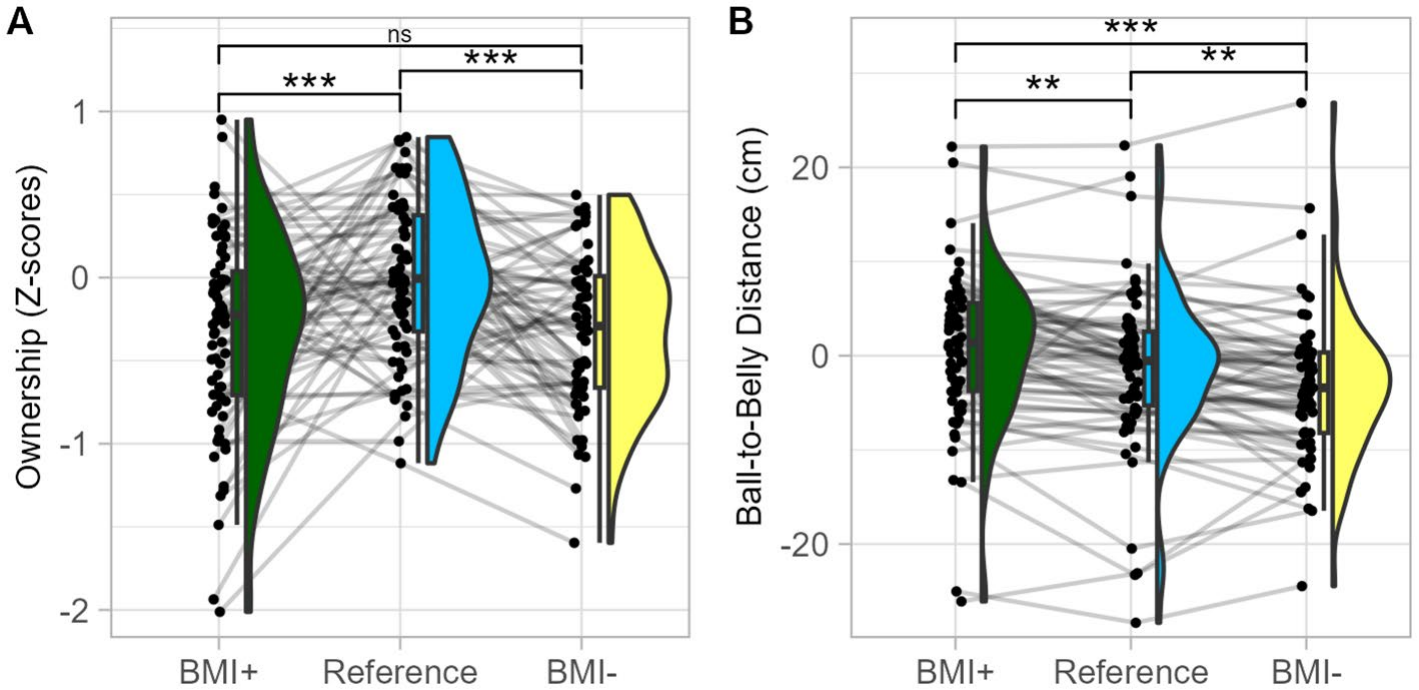
Effect of Avatar Body Mass Index in Experiment 2 (visuo-motor). Distribution of ownership ratings (**A**) and ball-to-belly distance (**B**) in the condition of avatar BMI + (green), Reference (blue) and BMI− (yellow). **p < .01; ***p < .001.

**BMI manipulation** Ownership feeling decreased in the condition of BMI + (p < 0.001) and BMI− (p < 0.001) avatar compared to the Reference avatar, with no difference between BMI + and BMI− avatar (p = 0.962) (Fig. 9.A). Post-hoc analysis on ball-to-belly distance revealed that, compared to the Reference avatar condition (mean intercept = − 1.24 cm), the ball-to-belly distance increased by 1.86 cm in the BMI + condition (p = 0.002), while it decreased by − 1.64 cm in the BMI− condition (p = 0.005). The difference between BMI + and BMI− was also significant (p < 0.001) (Fig. 9.B). This indicates embodiment of these avatars. For heart-rate variability, post-hoc analyses revealed no significant pairwise difference after correcting for multiple comparisons.

##### *Interaction Age* × *Avatar*

We did not observe any significant interaction between Age and Avatar on the explicit measures nor implicit measures nor physiological measures.

## Discussion

Adolescence is a crucial transition period for developing a healthy and stable sense of self. Despite the role of bodily self-consciousness in shaping other components of the self^11,47^, experimental evidence on how bodily self-consciousness may change during adolescence is scarce. The present study sought to fill this gap by using the full-body ownership illusion paradigm in girls aged 10–17. We tested whether the tendency to embody a virtual body with incongruent visuo-tactile or visuo-motor stimulation and incongruent appearance varied as a function of age. We found that the effect of visuo-tactile or visuo-motor asynchrony on agency or referral of touch increased with age, as did the effect of a discrepant avatar on ownership. Overall, this suggests that bodily self-consciousness becomes more robust with increasing age during adolescence and, consequently, that the ability to discriminate bodily-self from non-self information matures during adolescence. We discuss successively the effects of incongruent timing and incongruent visual appearance, noting that there was no significant interaction between the two types of manipulation.

### The effect of asynchronous stimulation on embodiment increases non-linearly during adolescence

Visuo-tactile and visuo-motor asynchrony are the most common types of multisensory incongruence used to study the mechanisms underlying embodiment over a body part or the full body. In adults, ownership, referral of touch or agency feelings decrease when the multisensory stimulation on the physical body does not temporally match the stimulation on the virtual body^14^. The main effects of visuo-tactile or visuo-motor asynchrony on four explicit measures reported in the present study confirm that asynchronous stimulation during full-body illusions is adapted to study bodily self-consciousness in adolescents.

Regarding ownership, previous research suggested that the effect of visuo-tactile^27^ or visuo-motor^26^ asynchrony reached adult’s level at around 10–13 years of age. However, no previous study has investigated changes in bodily self-consciousness between early and late adolescence. Our study fills this gap by showing that asynchrony between seen and felt tactile or motor stimulation decreases ownership feeling to the same extent in participants aged 10 to 17 years. Therefore, even if somatosensory integration mechanisms continue to mature during adolescence^48^, age does not seem to markedly impact the effect of asynchrony on ownership in full-body illusions.

When considering other components of bodily self-consciousness, conclusions differ, however. Our results suggest that the effect of visuo-tactile and visuo-motor asynchrony on referral of touch and agency increases from 10 to 17 years, with a significant increase at the beginning of adolescence followed by a plateau from 12 to 14 and then an increase again starting around 14–16 years. This age effect was also observed when looking at the onset time of the illusion: the older the participants, the more the illusion onset was delayed, with the delay becoming stable at around 15 years. There was a trend for the effect of visuo-motor asynchrony to follow a similar pattern. This is consistent with a previous study^26^ that reported that asynchronous avatar movement decreased agency more in adults than in individuals aged 8–12 years. Our study indicates that this developmental difference may still be present later in adolescence. The similar development of asynchrony effect over tactile and motor stimulations suggests that embodiment mechanisms are related to central multisensory integration abilities, rather than solely relying on unisensory processing^49^. Moreover, our data point to the age of 14–16 years as an important period, when discriminating self and non-self-related information regarding touch and actions improves. This is coherent with studies on the development of multisensory integration that showed that the ability to accurately perceive temporal relations between auditory and visual stimuli increases during childhood^50^ and until mid or late adolescence or adulthood^51^. However, studies on multisensory integration in adolescence are limited and rarely look at proprioception and further research is needed to better characterize this developmental pattern.

The different age effect on ownership and agency or referral of touch provides new insights on the relations between different aspects of bodily self-consciousness. By showing that, in asynchronous conditions, the age of teenagers modulates referral of touch and agency but not ownership feeling, our results support models that conceptualize ownership on one hand and referral of touch and agency on another hand, as different components of bodily self-consciousness^49,52^. The latter would reflect visuo-tactile or visuo-motor integration, which are not functionally mature at the beginning of adolescence^5^. Ownership feeling, in contrast, would be supported by causal inference processes beyond mere sensory integration^53^. These mechanisms could be already mature by the age of 10.

Our results showing that age influences referral of touch appear to be inconsistent with previous findings^27^ that indicated that the effect of visuo-tactile asynchrony on touch referral did not differ between 10–11 years old children and adults. This study used however third-person perspective, which implements spatial conflict between the participant’s own body, as sensed from the first-person perspective, and the avatar observed at a distance^54^. This decreases embodiment of the avatar and likely influences how multisensory integration produces the sense of corporeal self^55^. It might be that the perception of spatial conflict is mature in young adolescents while the perception of multisensory conflict is not.

### The effect of the avatar’s shape reveals that internal body representation changes during adolescence

How a discrepant avatar’s shape affects the illusion is not well understood, even in adults. Here, we used a personalized avatar and manipulated its pubertal status appearance. We observed that older adolescents have a stronger ownership feeling towards an Adult-like avatar, while younger adolescents have a stronger ownership feeling towards a Child-like avatar compared to their Reference avatar. This is consistent with the idea that individuals tend to embody avatar that more closely resemble their own appearance. Interestingly, ownership feeling towards the Reference avatar was stronger in older participants compared to young participants. This suggests that young adolescents have not yet fully integrated their own body shape and may have a somewhat blurred distinction between self and non-self morphological shapes, and overall, a more malleable bodily self-consciousness. Adolescence is characterized by a significant increase in growth rates and bodily changes^57^, which has consequences at the somatosensory-motor level^58^. Our results highlight that changes also occur for higher-level body representations. This is in line with clinical and psychoanalytical approaches putting forward that adolescence is conducive to the emergence of feelings of unfamiliarity with one’s own body^59^.

We did not observe any significant interaction between participant’s age and the reduction of ownership feeling due to increasing or decreasing the avatar’s body mass index. This suggests that younger adolescents do not feel more, or less, ownership than older adolescents for an avatar with a larger or smaller body mass index than their own. This finding is consistent with the hypothesis that high-level processes related to body morphology determine the ownership feeling^60^. This is in line with Provenzano et al.^61^^.^ study who manipulated avatar BMI with also + 15% and − 15% of BMI, and reported, in young adults, an increased ownership in the BMI + compared to reference avatar. This result is however in contradiction with other studies that reported that avatar shape manipulation did not affect the strength of subjective embodiment in adults^62^ nor in children^25^. It remains unclear how perspective and avatar characteristics for instance could explain the difference across studies^63^. A possibility is that adolescents and young adults, or boys and girls, differ in this matter, with adults or boys more familiar with bodies of larger size. Yet, more data is necessary to characterize the link between visual appearance and body ownership. In contrast to body ownership feeling, feelings of agency or referral of touch were not impacted by manipulating the avatar appearance, in line with the study of Provenzano et al.^61^. This suggests that ownership relies more on visual aspects of the body than agency or referral of touch and supports the differentiation between the processes underlying ownership, on a one hand, and referral of touch and agency, on the other hand.

The interaction effect of age and avatar pubertal appearance was significant in the case of visuo-tactile induction of embodiment but not in the case of visuo-motor induction. One explanation could come from a Bayesian account of body ownership^53^, which considers that the sense of body ownership is constructed by estimating the probability that various noisy sensory signals share a common cause, taking into account spatial proximity, simultaneity, uncertainty, and prior perceptual experiences. Interpreting our results under the lens of this model, we speculate that proprioceptive and/or motor information may have a more important weight than tactile information in determining ownership.

### Explicit and implicit measures: what are we measuring?

In our study, we asked participants to press a button when they estimated that a virtual ball touched their real body. Our results suggest that when embodying an avatar with a higher BMI, adolescents estimate their own body as wider and, conversely, when presented with an avatar with a lower BMI, they estimate it as thinner. This is coherent with previous studies reporting that after embodying a larger or thinner avatar, participants change the explicit estimation of their body size^64^ or their movements^65^. Whereas subjective ratings of ownership decrease when participants see an avatar with modified BMI, objective measures support the idea that participants still embody these avatars, at least enough to implicitly modify their body size and shape representation. Additionally, the implicit body size perception (ball-to-belly distance task) was impacted by the avatar’s BMI also when the stimulation was asynchronous. This suggests that participants could implicitly embody avatars even when there was a delay between the seen and felt stimulation, although this was not captured in the explicit ratings of ownership and agency/referral of touch. This reflects that different measures of embodiment allow investigating distinct levels of bodily self-consciousness^66^. In this vein, it could be noted that our ball-to-belly distance task, while assessing implicit body size representation, is also an indication of the expansion/shrinkage of the peri-personal space. The peri-personal space is the space surrounding the body that supports its interaction with the environment. Multisensory integration within the peri-personal space is at the basis of bodily self-consciousness^12^, and it is modulated by changes in body morphology like, for example, during pregnancy^56^. The fact there is no effect of age on the task and its modulation by the size of the avatar, indicates that the mechanisms underlying multisensory integration in peripersonal space are in place in adolescence. Yet, more work is needed to determine more comprehensively how peri-personal space develops.

Contrary to our hypothesis, we did not find a significant effect of the shape and size of the avatar on physiological measures. One possible explanation is that, additionally to age, other inter-individual factors, like for example body esteem, influence the physiological response to avatar modification. Another possibility is that the rest period between the different conditions was too short to completely erase the previous illusory bodily experience.

We found, however, an effect of visuo-motor asynchrony on three physiological measures. More precisely, the non-linear component of heart rate variability, the number and the amplitude of non-specific skin conductance responses were higher in the synchronous than in the asynchronous condition. These results are partly in line with a previous study that used a visuo-tactile rubber-hand illusion^22^ and reported that non-specific skin conductance peaks were higher in synchronous vs asynchronous conditions. This may be due to a stronger arousal in the visuo-motor synchronous condition compared to the asynchronous condition. The effect of avatar appearance manipulation on physiological measures was weaker and inconsistent in post-hoc analyses emphasizing that it is the action mismatch that leads to autonomic effects. This is consistent with participants’ informal reports of some degree of discomfort during asynchronous movements of the avatar. Also, the lack of asynchrony or avatar appearance effect on physiological measures in the visuo-tactile condition may indicate a stronger contribution of visuo-motor as compared to visuo-tactile information in shaping full-body embodiment experiences and bodily self-consciousness.

## Conclusion

Our study reveals that younger adolescents have a more malleable bodily self-consciousness than older peers. From 10 to 17 years of age, referral of touch and agency feelings become more robust. Also, ownership feeling becomes more impacted by visual information during adolescence. Considering that ownership is impacted by avatar size and shape whereas agency is impacted by asynchrony manipulation, our results support the idea that ownership, on one hand, and referral of touch and agency, on the other hand, rely on different mechanisms.

Our study complements previous ones that showed that mental and social aspects of self-experience are maturing throughout adolescence^6^. Future studies should explore how these different aspects of self-experience relate to each other, whether they evolve in parallel, together, or if the maturation of one precedes the maturation of others. This question is particularly relevant considering that many psychiatric disorders emerge during adolescence^67^ and considering the proposed role of sensory mechanisms^68^ and bodily self-consciousness^69^ in psychiatric disorders’ onset. Specifically, the onset of a major disorder of bodily self-consciousness, namely anorexia nervosa, peaks around 15 years^70^ and has been linked to multisensory integration deficits^71^. Also, anorexic patients have been reported to experience a stronger rubber hand illusion^72^ than healthy participants. It is possible that the reduction in malleability of bodily self-consciousness during adolescent development, as evidenced in our data in healthy individuals, could be altered in anorexia nervosa. This should be investigated more specifically in future studies. Also, we focused our study on adolescent girls precisely to allow, in the future, comparing them with individuals on anorexia nervosa as this condition predominantly affects females. It would be important to investigate changes in bodily self-consciousness in male participants as well. We speculate that results may be similar although a shift in age may be expected given the later development of boys.

### Supplementary Information

Supplementary Information.

## Data Availability

The dataset generated and analysed during the current study is available from the corresponding author on reasonable request. In addition, it will be made accessible on OSF upon publication.
