# Supplementary material for: Developmental changes of bodily self-consciousness in adolescent girls
Source: Sci Rep. 2024 May 17;14:11296. doi: 10.1038/s41598-024-61253-6 (PMC11101456; doi:10.1038/s41598-024-61253-6)
Supplement: Supplementary file 1 — Supplementary Information. [file 41598_2024_61253_MOESM1_ESM.docx]

Supplementary Information

**Developmental Changes of Bodily Self-Consciousness in Adolescents Girls**

Lisa Raoul^1^, Cédric Goulon^2^, Fabrice Sarlegna^2^, Marie-Hélène Grosbras ^1,∗^

# Supplementary Material and Methods

## Physiological data recording and preprocessing

We recorded cardiac (ECG) and electrodermal activity (EDA) using a BIOPAC MP160. The sampling rate was kept at 2000 samples per second.

For ECG recording, a three-lead configuration (Einthoven Triangle with Lower Left and right Ribs and Right Clavicle) was used. Raw signal was filtered using 0.5 Hz high-pass butterworth filter (order 5), followed by a notch filter to remove 50 Hz interference.

For EDA recording, two electrodes were positioned on the palmar surface of the second and third fingers of the left hand. Raw EDA signal was down-sampled to 250 Hz. Smoothing baseline method was used to compute phasic skin conductance and then subtracted from the raw waveform to obtain tonic skin conductance waveform. From this, non-specific skin conductance responses (SCRs) were identified as peaks above 0.001 µS with a rejection threshold of 10% of the participant’s largest peak. Amplitude values of SCRs were square-root-transformed to follow[^1^](#ref-dawson_electrodermal_2007) recommendations.

# Supplementary Results

## Detailed statistics

### Experiment 1: visuo-tactile induction of embodiment

#### Interactions between factors

Table S1: Interaction effects on the different measures in Experimen1 ( visuotactile); all non significant

| Measures | Asynchrony.Avatar | Age.Asynchrony.Avatar |
| --- | --- | --- |
| Occurrence | X²(4)=5.74 | X²(4)=0.49 |
|  | p=0.22 | p=0.974 |
| Onset Time | F(4,462)=1 | F(4,462)=1.28 |
|  | p=0.408 | p=0.277 |
| Ownership | F(4,607)=0.03 | F(4,607)=0.24 |
|  | p=0.998 | p=0.914 |
| Ref Touch | F(4,607)=0.94 | F(4,607)=0.62 |
|  | p=0.442 | p=0.647 |
| Ball-Belly Dist | F(4,579)=0.51 | F(4,579)=0.97 |
|  | p=0.728 | p=0.424 |
| HRV RMSSD | F(4,543)=0.9 | F(4,543)=0.89 |
|  | p=0.464 | p=0.47 |
| HRV HF | F(4,543)=1.26 | F(4,543)=2.11 |
|  | p=0.284 | p=0.078 |
| HRV SD2 | F(4,543)=0.5 | F(4,543)=0.57 |
|  | p=0.736 | p=0.682 |
| SCR Amplitude | F(4,430)=1.38 | F(4,430)=0.55 |
|  | p=0.24 | p=0.7 |
| SCR Number | F(4,430)=1.51 | F(4,430)=0.46 |
|  | p=0.197 | p=0.768 |

#### Effect of Asynchrony

##### Main effect- Comparison synchronous *vs* asynchronous condition

probability of illusion occurrence : beta = -4.49, CI = [-5.45,-3.53], z = -9.16, p < 0.001

onset time beta=0.89, CI95=[0.74,1.05], p<0.001

ownership ratings beta=-0.39, CI95=[-0.48,-0.3], p<0.001

referral of touch ratings beta=-1.07, CI95=[-1.2,-0.93], p<0.001

##### Interaction Age X Asynchrony

Effect of age^2^ on onset time

in the synchronous condition : F(2,66) = 0.026, p = .97

in the asynchronous F(2,54) = 6.54, p = 0.003

Effect of age ^3^ on referral of touch ratings

in the synchronous condition : F(3,66) = 1.15, p = .33)

in the asynchronous condition F(23,66) = 3.16, p = 0.030.

#### Effect of Avatar

##### Main effect

**Maturational shape manipulation.**

Ownership:

adult-like vs reference: beta = 0.1, CI95= [-0.04,0.24], p=0.209

child-like vs reference : beta = 0.16, CI95= [0.02,0.31], p=0.056.

adult-like vs and child-like : beta = 0.06, CI95= [-0.08,0.21], p=0.402

Ball-to-belly distance

adult-like vs reference: beta = -1.29, CI95= [-2.32,-0.26], p=0.021

child-like vs reference : beta = 0.29, CI95= [-0.74,1.32], p=0.58

adult-like vs child-like : beta = 1.58, CI95= [0.54,2.61], p=0.006

**BMI manipulation.**

Ownership

BMI+ vs reference: beta = 0.23, CI95= [0.09,0.38], p=0.004

BMI- vs reference: beta = 0.34, CI95= [0.2,0.48], p<0.001

BMI+ vs BMI- :beta = -0.1, CI95= [-0.25,0.04], p=0.209

Ball-to-belly distance

BMI+ vs reference : beta = -2.34, CI95= [-3.37,-1.32], p<0.001

BMI- vs reference : beta = 1, CI95= [-0.03,2.02], p=0.068

BMI+ vs BMI- : beta = -3.34, CI95= [-4.37,-2.31], p<0.001

##### Interaction Age X Avatar

**Post-hoc analyses for Interaction Age x Maturational shape manipulation.**

ownership feeling

effect of age for the reference avatar : beta = 0.07, CI95= [0.01,0.13],p=0.018

effect of age for the adult-like avatar : beta = 0.1, CI95= [0.04,0.15],p=0.001

effect of age for the child-like avatar : beta = -0.01, CI95= [-0.07,0.05],p=0.798

child-like vs reference : beta = 0.084, CI95= [0.012,0.144], p=0.036

child-like vs adult-like : beta = 0.108, CI95= [0.036,0.168], p=0.007

adult-like vs reference : beta = -0.024, CI95= [-0.096,0.048], p=0.593

**Post-hoc analyses for Interaction Age x BMI manipulation.**

effect of age for BMI+ avatar : beta = 0.07, CI95= [0.01,0.13],p=0.036

effect of age for the BMI- avatar : beta = 0.02, CI95= [-0.05,0.08],p=0.622

BMI- vs reference : beta = 0.06, CI95= [-0.012,0.12], p=0.154

BMI+ vs reference : beta = 0, CI95= [-0.06,0.072], p=0.886

### Experiment 2 with visuo-motor induction of embodiment.

*Interactions*

Table S2: Interaction effects on the different measures in Expeirment 2 ( visuo-motor)

| Measures | Asynchrony.Avatar | Age.Asynchrony.Avatar |
| --- | --- | --- |
| Occurrence | X²(4)=2.52 | X²(4)=3.12 |
|  | p=0.641 | p=0.538 |
| Onset Time | F(4,291)=0.22 | F(4,291)=0.37 |
|  | p=0.925 | p=0.833 |
| Ownership | F(4,596)=0.51 | F(4,596)=1.7 |
|  | p=0.727 | p=0.148 |
| Agency | F(4,595)=0.57 | F(4,595)=0.46 |
|  | p=0.685 | p=0.768 |
| Ball-Belly Dist | F(4,575)=1.88 | F(4,575)=1.92 |
|  | p=0.113 | p=0.105 |
| HRV RMSSD | F(4,537)=1.47 | F(4,537)=0.57 |
|  | p=0.21 | p=0.682 |
| HRV HF | F(4,537)=1.5 | F(4,537)=0.65 |
|  | p=0.2 | p=0.627 |
| HRV SD2 | F(4,537)=0.27 | F(4,537)=1.1 |
|  | p=0.895 | p=0.353 |
| SCR Amplitude | F(4,430)=0.53 | F(4,430)=0.7 |
|  | p=0.713 | p=0.591 |
| SCR Number | F(4,430)=1.51 | F(4,430)=0.26 |
|  | p=0.199 | p=0.901 |

#### Effect of Asynchrony

##### Main effect Comparison synchronous *vs* asynchronous condition

probability of illusion occurrence : beta = -3.95, CI = [-4.52,-3.38], z = -13.54, p < 0.001

onset time : beta=0.85, CI95=[0.61,1.08], p<0.001

ownership : beta=-0.95, CI95=[-1.04,-0.85], p<0.001

agency : beta=-1.19, CI95=[-1.31,-1.07], p<0.001

hear-rate variability non-linear component (SD2 index) : beta=3.78, CI95=[1.45,6.12], p=0.002)

number of peaks in skin conductance : beta=-0.96, CI95=[-1.46,-0.46], p<0.001

amplitude of peaks in skin conductance : beta=-0.02, CI95=[-0.04,0], p=0.029

##### Interaction Age X Asynchrony

Effect of age on agency ratings

in the synchronous condition : F(3,65) = 2.73,p = 0.051

in the asynchronous condition : F(3,65) = 2.30, p = 0.037

#### Effect of Avatar

##### Main effect

**Maturational shape manipulation.**

ownership feeling

adult-like vs reference: beta = 0.14, CI95= [-0.01,0.29], p=0.104

child-like vs reference: beta = 0.19, CI95= [0.04,0.35], p=0.023

child-like vs adult-like: beta = 0.05, CI95= [-0.1,0.21], p=0.578

ball-to-belly distance

adult-like vs reference: beta = -0.86, CI95= [-1.93,0.2], p=0.135

child-like vs reference : beta = 0.03, CI95= [-1.04,1.09], p=0.962

child-like vs adult-like: beta = 0.89, CI95= [-0.18,1.95], p=0.135

heart-rate variability

adult-like vs reference: beta = -3.54, CI95= [-7.21,0.13], p=0.175

child-like vs reference :beta = -4.16, CI95= [-7.82,-0.5], p=0.155

child-like vs adult-like: beta = -0.62, CI95= [-4.29,3.05], p=0.81

**BMI manipulation.**

ownership feeling

BMI+ vs reference : beta = 0.33, CI95= [0.18,0.48], p<0.001

BMI- vs reference : beta = 0.32, CI95= [0.17,0.47], p<0.001

BMI+ vs BMI- :beta = 0, CI95= [-0.15,0.15], p=0.962

ball-to-belly distance

BMI + vs reference: beta = -1.87, CI95= [-2.93,-0.81], p=0.002

BMI- vs reference : beta = 1.64, CI95= [0.58,2.7], p=0.005

BMI+ vs BMI- : beta = -3.51, CI95= [-4.57,-2.45], p<0.001

heart-rate variability HRV_RMSSD:

BMI+vs reference : beta = 0.45, CI95= [-3.2,4.1], p=0.81

BMI- vs reference : beta = -1.18, CI95= [-4.83,2.47], p=0.789

BMI+ vs BMI- : beta = 1.62, CI95= [-2.02,5.27], p=0.764

HRV_HF :

BMI+ vs reference: beta = 187.97, CI95= [-39.18,415.12], p=0.412

BMI- vs reference: beta = 73.02, CI95= [-154.12,300.17], p=0.528

BMI+vs BMI- (beta = 114.95, CI95= [-112.2,342.09], p=0.528

## Results from raw data analyses in Experiment 1 with visuo-tactile induction of embodiment (Raw onset time and subjective report)

Analyses on raw onset time and subjective reports showed no significant interaction between the factors Asynchrony and Avatar, nor a three-way interaction between Age, Asynchrony, and Avatar. Therefore, models without these interaction terms are reported. Statistics for main and interaction effects on non-transformed data are reported in Table S1.

| Measures | Asynchrony | Avatar | Age.Asynchrony | Age.Avatar |
| --- | --- | --- | --- | --- |
| Onset Time | **F(1,483)=90.78** | F(4,468)=0.27 | **F(2,481)=18.22** | F(4,469)=0.34 |
|  | **p<0.001** | p=0.895 | **p<0.001** | p=0.851 |
| Ownership | **F(1,615)=69.89** | **F(4,615)=5.4** | F(1,615)=1.25 | **F(4,615)=4.48** |
|  | **p<0.001** | **p<0.001** | p=0.264 | **p=0.001** |
| Ref Touch | **F(1,613)=210.96** | F(4,613)=0.05 | **F(3,613)=24.48** | F(4,613)=1.9 |
|  | **p<0.001** | p=0.996 | **p<0.001** | p=0.11 |

*Table S1: Main and interaction effects on raw data in Experiment 1; For the interaction Age x Asynchrony, values refer to models with quadratic (for onset time) and cubic (for reference of touch) age term.*

### Effect of Asynchrony

#### Main effect of Asynchrony

Onset time was higher (beta=17.52, CI95=[13.94,21.07], p<0.001), and ownership ratings (beta=-13.14, CI95=[-16.2,-10.08], p<0.001), as well referral-of-touch ratings (beta=-32.72, CI95=[-37.1,-28.34], p<0.001), were lower in asynchronous compared to synchronous conditions (see Figure S 1)


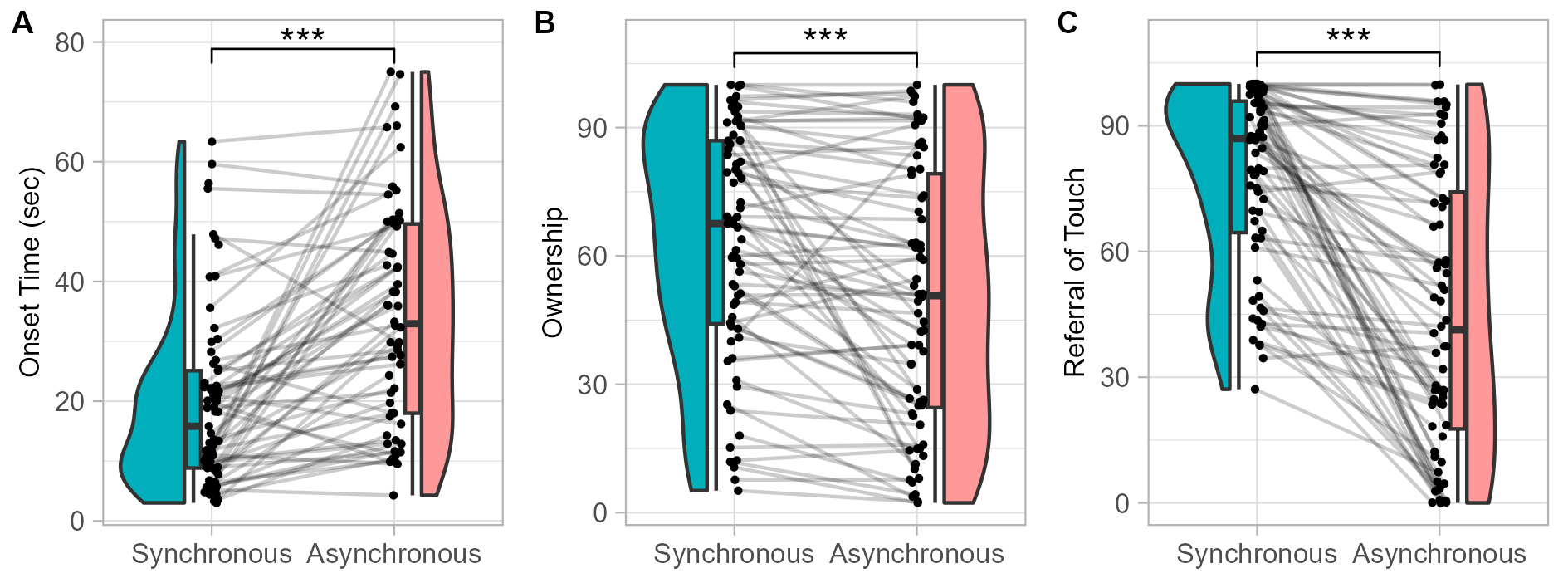


*Figure S1 Effect of Asynchrony on explicit measures in Experiment 1 (Visuo-Tactile). Distribution of raw onset times (A), raw ownership (B) and raw referral of touch (C) ratings in synchronous (blue) and asynchronous (red) trials. ’***’: p < .001*

#### Interaction of Age and Asynchrony

Moreover, we observed an interaction between the effect of Asynchrony and Age on onset time and on Referral of Touch (Figure S 2).


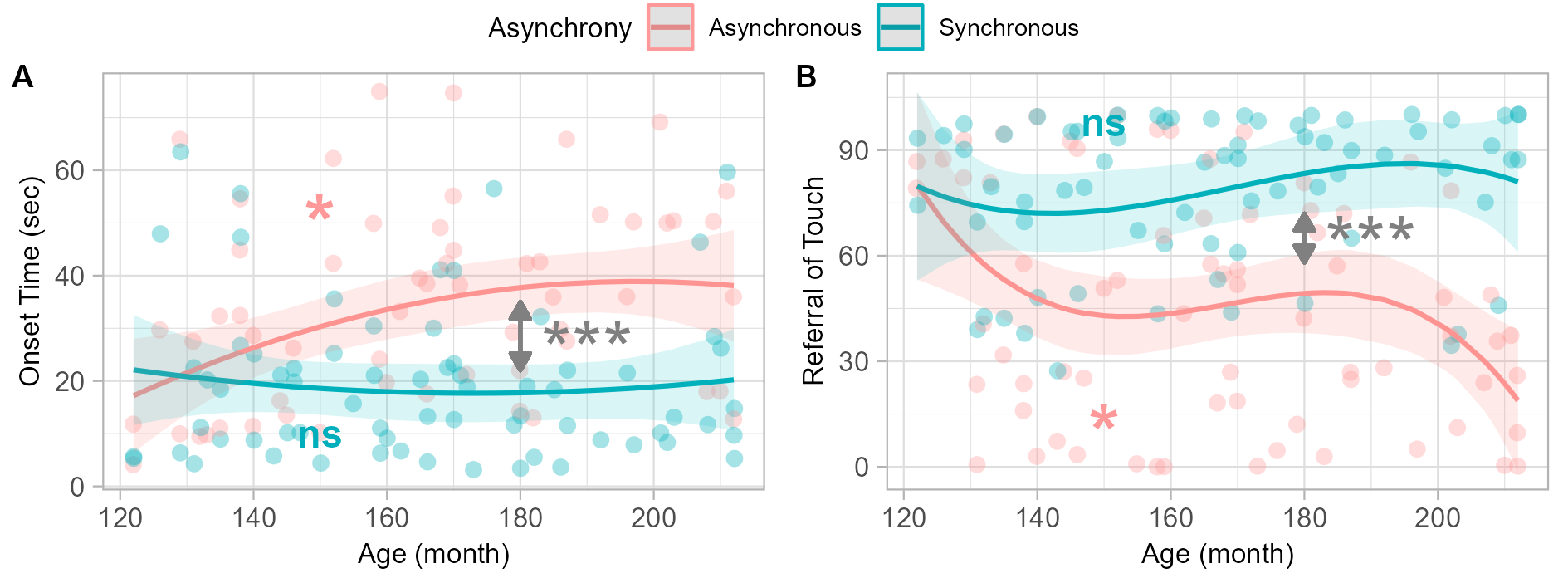


*Figure S2 Interaction Age x Asynchrony on explicit measures in Experiment 1 (Visuo-Tactile) , with the 95 percent confidence interval indicated by shaded areas. Dots represent individual’s mean rating over the 5 avatar appearance trials (as there is no interaction AvatarxAsynchrony). ’**‘: p < .001;’*‘: p < .05; ’ns’: > 0.10*

For onset time, this was better described by a quadratic relationship (X²(2)=8.43, p=0.015). Post-hoc analyses showed that the difference between synchronous and asynchronous conditions increases non-linearly with increasing age. Looking at it the other way, there was no effect of age on the onset time of the illusion in the synchronous condition (F(2,66) = 0.26, p = .76) but only in the asynchronous condition, with onset time increasing non-linearly with increasing age (F(2,54) = 4.15, p = 0.021). The inflection point occurred at 193 months ($\approx$ 16.1years), after which there was no noticeable change with age.

For referral-of -touch ratings, the interaction between the effect of asynchrony and age was better described by a cubic relationship (X²(4)=11.28, p=0.024). Post-hoc analyses showed that the difference between synchronous and asynchronous conditions increases non-linearly with age. There was no effect of age in the synchronous condition (F(3,66) = 0.88, p = .45) but only in the asynchronous condition, with referral-of-touch ratings decreasing non-linearly with increasing age (F(23,66) = 2.87, p = 0.043). Analysis of the derivative of this function showed a steep decrease from 120 to 153 months ($\approx$ 12.8 years), followed by a plateau until 181 months ($\approx$ 15.1 years) before decreasing.

### Effect of Avatar

#### Main effect of Avatar

Regarding the explicit measures, manipulating the avatar’s shape impacted only the ratings of ownership feeling (Figure S3).


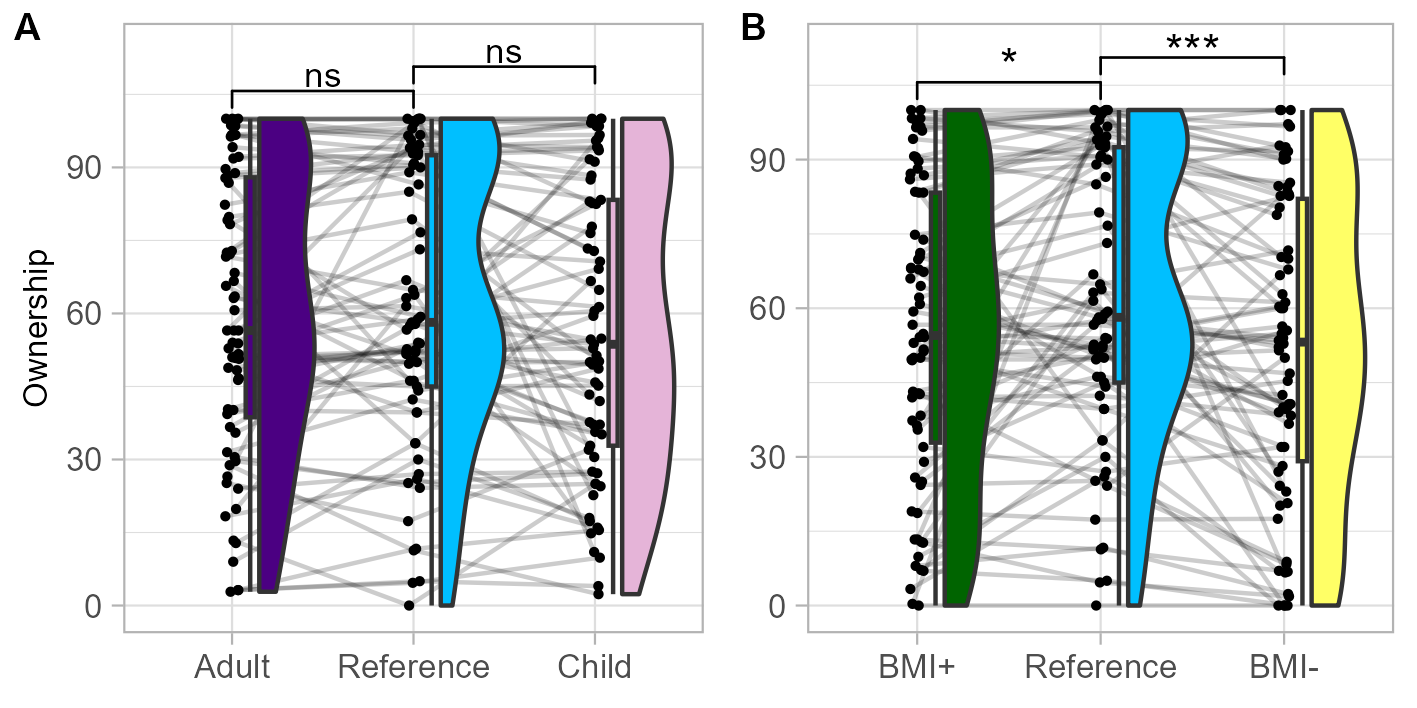


*Figure S3 Effect of the avatar’s appearance in Experiment 1 (Visuo-Tactile). Distribution of raw ownership ratings for (A) maturational shape manipulation [Adult-like(Purple), Reference (blue) and Child-like (pink) avatars] and (B) Body Mass Index Manipulation [BMI+(green), Reference (blue) and BMI- (yellow) avatars]. ’’: p < .001; ’’: p < .01; ’’: p < .05; ‘.’: p < .10; ‘ns’: > 0.10*

Post-hoc analyses were performed separately for maturational shape and BMI manipulations. Results are the same as those observed when analyzing Z-scores.

**Maturational shape manipulation.** The difference in ownership ratings was not significant when comparing the reference avatar with the adult-like (beta = 5.07, CI95= [0.18,9.96], p=0.112) or child-like avatars (beta = 3.04, CI95= [-1.86,7.94], p=0.251). The difference between adult-like and child-like avatars was also not significant (beta = 2.03, CI95= [-2.87,6.92], p=0.402) (Figure S3.A).

**BMI manipulation.** Ownership feeling decreased in the condition BMI+ (beta = 7.61, CI95= [2.73,12.49], p=0.012) and BMI- (beta = 10.66, CI95= [5.77,15.54], p<0.001) compared to the reference avatra. The difference between BMI+ and BMI- was not significant (beta = -3.05, CI95= [-7.91,1.81], p=0.251)(Figure 3.B).

#### Interaction of Age and Avatar

Moreover, as with Z-scores, we observed an interaction between the effect of Avatar and Age on ownership ratings, but not on the other explicit measures. A post-hoc analysis revealed no significant effect of age on ownership for the reference avatar (F(1,67) = 2.1,p = 0.14).


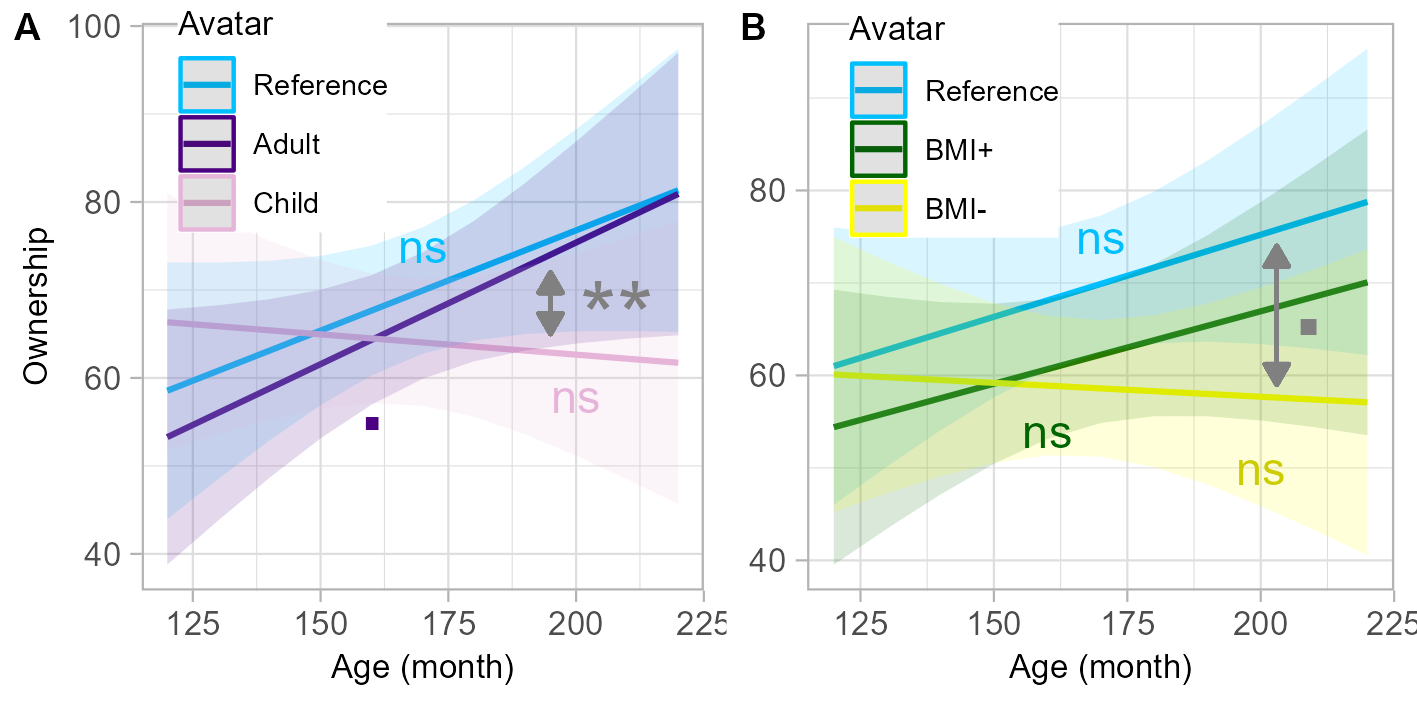


*Figure S4. Interaction Age x Avatar on raw ownership ratings in Experiment 1 (Visuo-Tactile). Lines represent the linear fit of the data as a function of age for maturational shape (A) and Body Mass Index (B) avatar appearance manipulation, with the 95 percent confidence interval indicated by shaded areas. Panel A represents the fits for Adult (Purple), Reference (blue) and Child (pink) avatars. Panel B represents the fits for avatar BMI+ (green), Reference (blue) and BMI- (yellow) avatars. ’**‘: p < .01;’.’: p < .01; ‘ns’: > 0.10*

**Maturational shape manipulation.**

Further post-hoc analyses of the interaction effect revealed that age had no significant effect on ownership ratings for the adult-like (F(1,68) = 2.87, p = 0.095) neither for the child-like avatars (F(1,68) = 0.50, p = 0.48). Pairwise comparisons (FDR corrected for 5 comparisons) showed that the difference in ownership ratings between the reference avatar and the child-like avatar increased with age (beta = 3.264, CI95= [1.032,5.508], p=0.011). There was no significant effect of age on the difference in ownership ratings between the reference and the adult-like avatars (beta = -0.612, CI95= [-2.856,1.62], p=0.737). The difference in ownership ratings between the child-like and the adult-like avatars increased with age (beta = 3.888, CI95= [1.656,6.12], p=0.003) (Figure S 4.A)

**BMI manipulation.** Moreover, age had no significant effect in the BMI+ (beta = 1.77, CI95= [-1.42,4.96],p=0.282) nor in the BMI- condition (beta = -0.48, CI95= [-3.76,2.81],p=0.777). Pairwise comparisons (FDR corrected for 5 comparisons) showed no significant effect of age on the difference in ownership rating between the reference and BMI- avatars (beta = 2.472, CI95= [0.24,4.716], p=0.05) neither between the reference and the BMI+ (beta = 0.24, CI95= [-2.004,2.472], p=0.837) or BMI+ and BMI- (beta = -3.05, CI95= [-7.91,1.81], p=0.251) avatars (Figure S4.B).

## Results from raw data analyses in Experiment 2 with visuo-motor induction of embodiment

Table S2: Main and interaction effects on raw data in Experiment 2; For the interaction Age x Asynchrony, values for ownership and agency refer to models with a quadratic age term

Likelihood ratio test show that model with quadratic age term in interaction with synchrony was better than linear term to predicted ownership (X²(6)=18.04, p=0.006) and agency (X²(2)=36.77, p<0.001).

| Measures | Asynchrony | Avatar | Age.Asynchrony | Age.Avatar |
| --- | --- | --- | --- | --- |
| Onset Time | **F(1,335)=47.91** | F(4,295)=1.77 | F(1,337)=1.27 | F(4,295)=2.15 |
|  | **p<0.001** | p=0.135 | p=0.26 | p=0.074 |
| Ownership | **F(1,603)=355.41** | **F(4,603)=5.69** | **F(1,603)=11.12** | F(4,603)=0.75 |
|  | **p<0.001** | **p<0.001** | **p=0.001** | p=0.556 |
| Agency | **F(1,602)=262.85** | F(4,602)=0.82 | **F(2,602)=57.87** | F(4,602)=0.22 |
|  | **p<0.001** | p=0.512 | **p<0.001** | p=0.928 |

### Effect of Asyncrhony

#### Main effect of Asynchrony

We observed a main effect of Asynchrony on all our explicit measures of illusion (Figure S5). Onset Time was higher (beta=18.43, CI95=[-33.75,31.46], p<0.001) and ownership ratings (beta=-32.19, CI95=[-35.51,-28.87], p<0.001), as well as agency ratings (beta=-34.36, CI95=[-38.48,-30.23], p<0.001), were lower in asynchronous than in synchronous conditions.


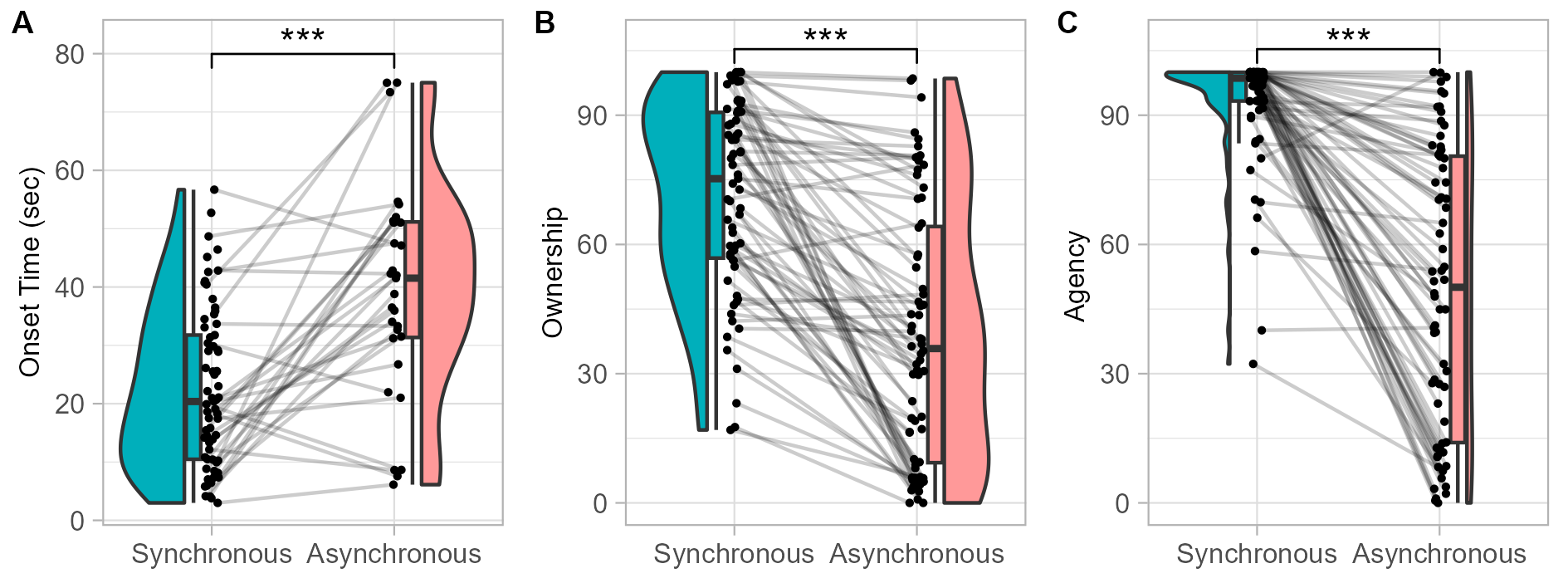


Figure S5:

*Effect of Asynchrony on raw explicit measures in Experiment 2 (Visuo-Motor). Distribution of raw onset times (A), raw ownership (B) and raw referral-of-touch (C) ratings in synchronous (blue) and asynchronous (red) conditions.*

We also observed an effect of Asynchrony on physiological measures, namely non-linear component of heart-rate variability and number and amplitude of skin conductance responses (Figure S6).


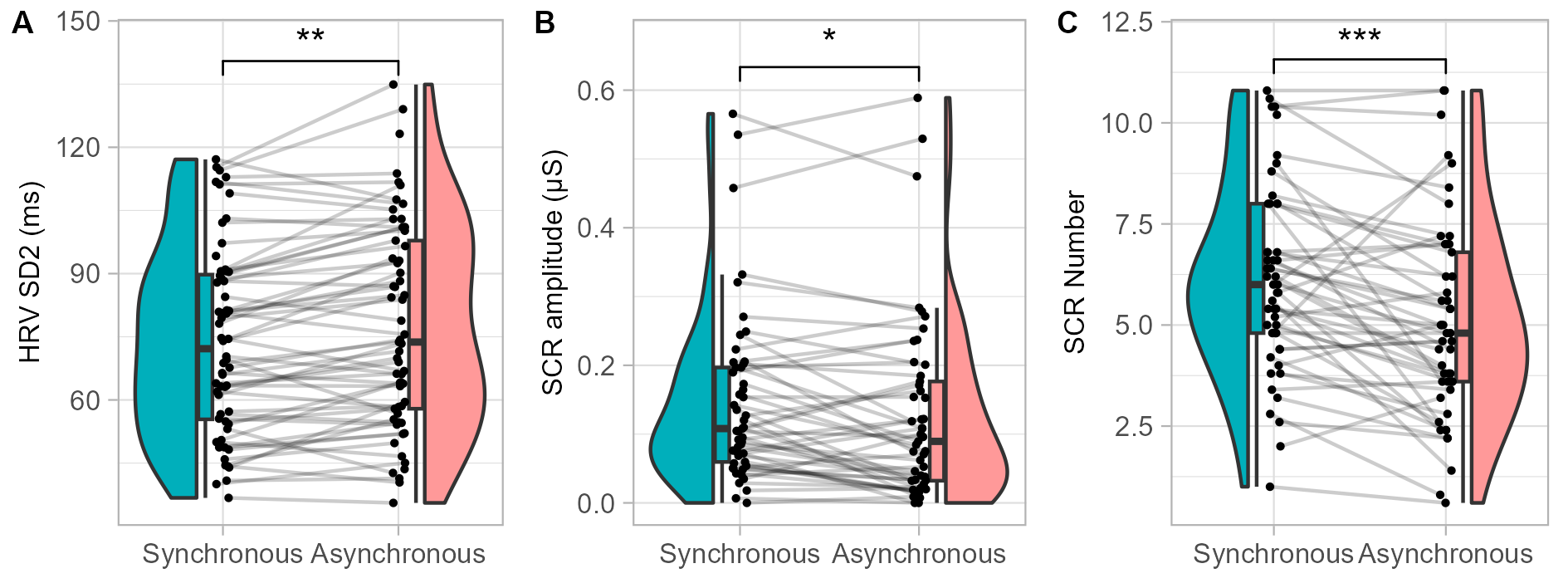


*FigureS6 Effect of Asynchrony on physiological measures in Experiment 2 .Distribution of HRV SD2 (A), SCR ampltidue (B) and SCR number (C) in synchronous (blue) and asynchronous (red) trials.*

#### Interaction of Age and Asynchrony

Moreover, we observed an interaction between the effect of Asynchrony and Age on raw ownership (Figure S7.A).The interaction was better described by a quadratic relation. Post-hoc analyses on ownership show that the difference between the synchronous and the asynchronous condition increases non linearly with age. However, when analyzing the two conditions separately, the effect of age was no significant in the synchronous condition (F(2,66) = 0.81,p = 0.45) nor in the asynchronous condition (F(2,66) = 1.45,p = 0.24). We observed an interaction between the effect of Asynchrony and Age on raw agency ratings (Figure S7.B).


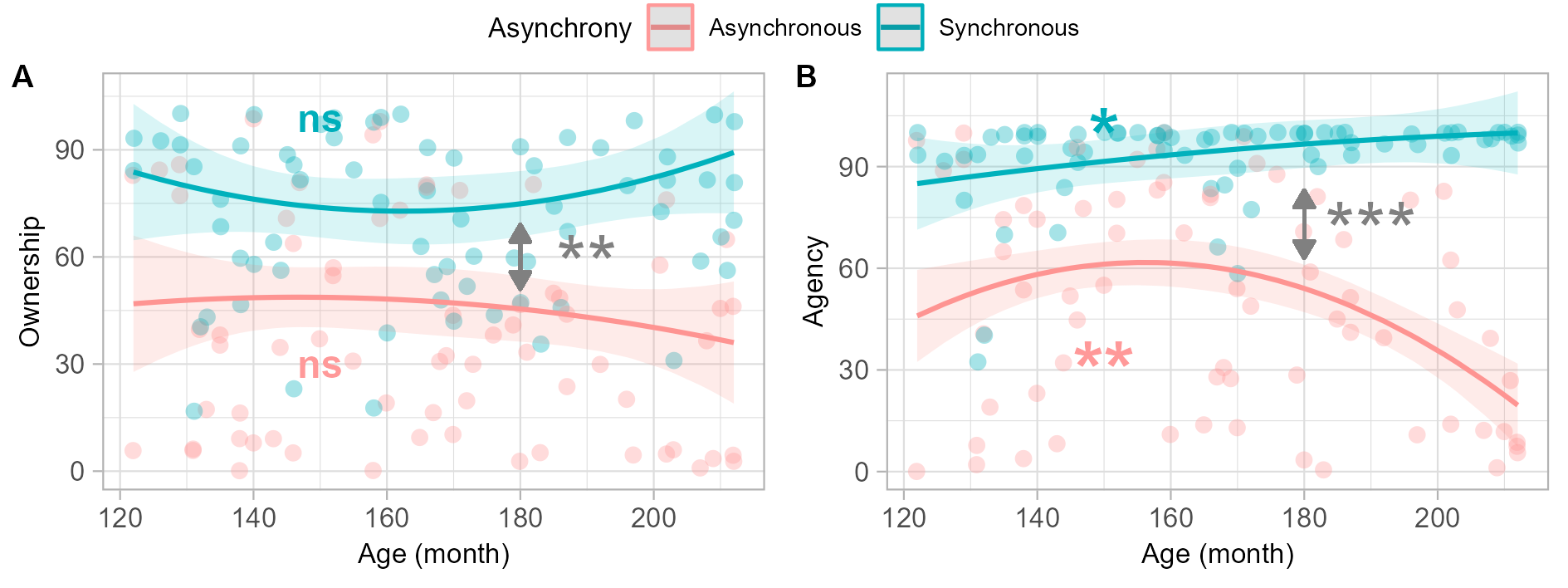


*Figure S7 Interaction Age x Asynchrony on explicit measures in Experiment 2 (Visuo-Motor). Lines represent the polynomial fit of agency ratings in synchronous (blue) and asynchronous (red) trials, with the 95 percent confidence interval indicated by colored areas. Dots represent individual mean rating over the 5 avatar appearance trials (as there is no interaction avatar*asynchrony).*

The interaction was better described by a quadratic model relationship. Post-hoc analyses showed that the difference between the synchronous condition and the asynchronous condition increased non linearly with age. Looking at it the other way, the effect of age in the synchronous condition was small with agency increasingly slightly with age (F(2,66) = 3.72, p = 0.029), whereas it showed a quadratic shape in the asynchronous condition with agency decreasing non linearly with increasing age (F(2,66) = 5.93, p = 0.005).

### Effect of Avatar

#### Main effect of Avatar

As for the analyses of Zscores presented in the main text, for the explicit measures, the manipulation of avatar shape impacted only the ratings of ownership feeling (Figure S 8). It did not impact the onset time nor the feeling of agency.


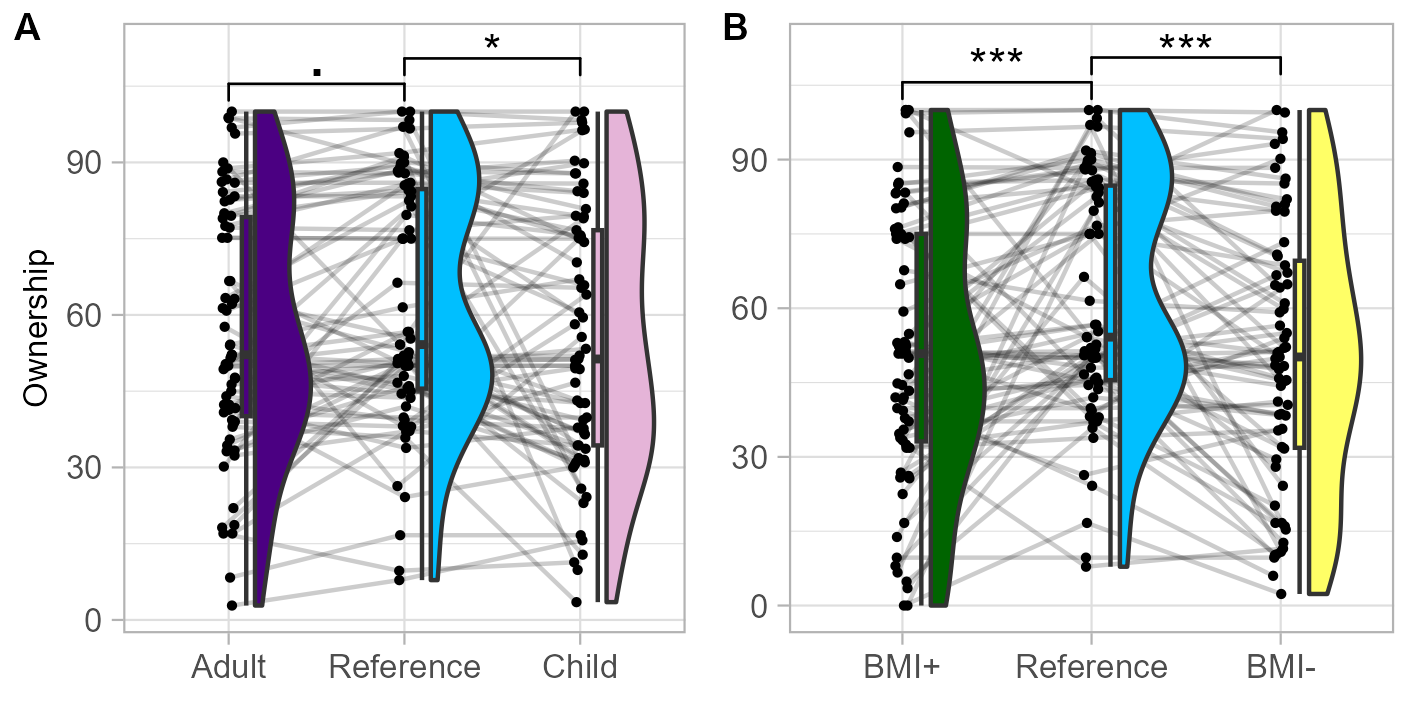


*Figure S8. Effect of Avatar on explicit and implicit measures in Experiment 2*

**Maturational shape manipulation.** When presented with an avatar with child-like features participants reported a weaker feeling of ownership than when presented with the reference avatar (beta = 6.39, CI95= [1.09,11.69], p=0.036). The difference in ownership rating was close to significance for the adult-like avatar (beta = 5.32, CI95= [0,10.64], p=0.075) (Figure 8.A).

**BMI manipulation.** Ownership feeling decreased in the condition of BMI+ (beta = 10.75, CI95= [5.45,16.04], p<0.001) and BMI- (beta = 11.22, CI95= [5.91,16.53], p<0.001) avatars compared to the reference avatar (Figure 8.B).

#### Interaction of Age and Avatar

Whereas the interaction between the effect of Avatar and Age on onset time was not significant when looking at the transformed onset time, it reached significance with raw onset time. However, post-hoc analyses revealed no effect of age on onset time in the conditions Reference (F(2,65 = 0.11, p = 0.89), child-like (F(1,67) = 1.24, p = 0.27), adult-like (F(1,66) = 0.165, p = 0.69), BMI+ a(F(1,67) = 1.80, p = 0.69) or BMI- (F(1,67) = 1.10, p = 0.29) avatars. They were also no meaningful interaction regarding the pairwise comparisons of interest.

# References

1. Dawson, M. E., Schell, A. M. & Filion, D. L. in *Handbook of Psychophysiology* (Cambridge University Press, 2007).
